# Domain adaptive deep possibilistic clustering for EEG-based emotion recognition

**DOI:** 10.3389/fnins.2025.1592070

**Published:** 2025-07-23

**Authors:** Yufang Dan, Qun Li, Xianhua Wang, Di Zhou

**Affiliations:** ^1^Institute of Artificial Intelligence Application, Ningbo Polytechnic, Ningbo, China; ^2^Ningbo Key Laboratory of Aging Health Equipment and Service Technology, Ningbo, China; ^3^Dazhou City Key Laboratory of Multidimensional Data Perception and Intelligent Information Processing, Dazhou, Sichuan, China; ^4^Sichuan Provincial Engineering Research Center of Meteorological Photoelectric Sensing Technology and Applications, Dazhou, Sichuan, China; ^5^Special Polymer Materials for Automobile Key Laboratory of Sichuan Province, Sichuan University of Arts and Science, Dazhou, China; ^6^Industrial Technological Institute of Intelligent Manufacturing, Sichuan University of Arts and Science, Sichuan, China

**Keywords:** electroencephalography, emotion recognition, deep domain adaptation, clustering assumption, memory bank

## Abstract

Emotion recognition based on electroencephalogram (EEG) faces substantial challenges. The variability of neural signals among different subjects and the scarcity of labeled data pose obstacles to the generalization ability of traditional domain adaptation (DA) methods. Existing approaches, especially those relying on the maximum mean discrepancy (MMD) technique, are often highly sensitive to domain mean shifts induced by noise. To overcome these limitations, a novel framework named **D**omain **A**daptive **D**eep **P**ossibilistic **c**lustering (**DADPc**) is proposed. This framework integrates deep domain-invariant feature learning with possibilistic clustering, reformulating Maximum Mean Discrepancy (MMD) as a one-centroid clustering task under a fuzzy entropy-regularized framework. Moreover, the DADPc incorporates adaptive weighted loss and memory bank strategies to enhance the reliability of pseudo-labels and cross-domain alignment. The proposed framework effectively mitigates noise-induced domain shifts while maintaining feature discriminability, offering a robust solution for EEG-based emotion recognition in practical applications. Extensive experiments conducted on three benchmark datasets (SEED, SEED-IV, and DEAP) demonstrate the superior performance of DADPc in emotion recognition tasks. The results show significant improvements in recognition accuracy and generalization capability across different experimental protocols, including cross-subject and cross-session scenarios. This research contributes to the field by providing a comprehensive approach that combines deep learning with possibilistic clustering, advancing the state-of-the-art in cross-domain EEG analysis.

## 1 Introduction

In the field of affective computing (Muhl C. and G., 2014), automatic emotion recognition (AER) (Stern, 2002) has gained significant attention (Kim et al., 2013; Zhang et al., 2013), especially for EEG-based emotion recognition (Wenming, 2017; Li et al., 2018a; Pandey and Seeja, 2019; Jenke et al., 2014; Musha T. and A., 1997). From a machine learning perspective, EEG-based AER tasks are typically formulated as classification or regression problems (Kim et al., 2013; Zhang et al., 2013). However, due to inter-subject variability in emotional expression patterns (Pandey and Seeja, 2019), classifiers trained on specific subjects often have poor generalization ability. Although optimizing feature representations and learning models has improved recognition accuracy (Li et al., 2018, 2019; Du et al., 2022; Song et al., 2018; Zhong et al., 2020; Zheng and Lu, 2015; Wei et al., 2015; Zhou et al., 2023), applying these classifiers to new subjects still yields unsatisfactory results (Zheng and Lu, 2013; Ghifary et al., 2017; Lan et al., 2019; Vinay et al., 2015; Wang et al., 2023). Domain Adaptation (DA) has emerged as a solution, aiming to transfer knowledge from related source domains to the target domain with scarce labeled data (VM Patel, 2015; Dan et al., 2022; Tao et al., 2017; Zhang et al., 2019; Tao et al., 2022).

The key to effective knowledge transfer in DA is to ensure data distribution similarity between the source domain and the target domain. Existing DA approaches mainly focus on distribution matching (such as instance re-weighting and feature mapping) and classifier model adaptation (Pan and Yang, 2018; VM Patel, 2015; Pan et al., 2011; Gretton et al., 2009; Chu et al., 2013; Long et al., 2013; Mahsa et al., 2013; Ganin et al., 2016; Kang et al., 2022; Liang et al., 2019; Tao et al., 2019, 2012, 2015, 2016). Early methods for addressing domain distribution shift, such as instance weighting techniques, including the popular Maximum Mean Discrepancy (MMD) (Gretton et al., 2009), have limitations. MMD often decouples optimization from classifier training. Feature mapping approaches (Pan et al., 2011; Long et al., 2013; Mahsa et al., 2013; Kang et al., 2022), like Transfer Component Analysis (TCA) (Pan et al., 2011) and Joint Domain Adaptation (JDA) (Long et al., 2013), have been developed to address these issues, but they still have drawbacks. Mahsa et al. (2013) introduced the Domain Invariant Projection (DIP) algorithm, which employs a polynomial kernel on the MMD metric to establish a concise shared feature space and decrease intra-class dispersion through a clustering-based method.

Conventional MMD-based DA approaches overlook the statistical framework of the target domain, which can impede accurate label prediction. Some methods, including the Contrastive Adaptation Network and Domain Invariant Projection Ensemble (Kang et al., 2022) attempt to address this issue, yet they remain MMD-based. Moreover, current MMD-based methods do not fully account for intra-domain noise, which can lead to mean-shift issues and compromise generalization.

Possibilistic clustering frameworks (Dan et al., 2024; Krishnapuram and Keller, 1993) offer a solution to these problems as they can mitigate noise interference during data clustering. The conventional MMD metric has been adapted into a single-cluster center objective in a noisy context, and a resilient domain adaptation classifier (EDPC) (Dan et al., 2024) based on the possibilistic distribution distance metric has been proposed. However, EDPC, as a shallow DA method, has limited feature extraction capabilities. Deep neural networks, with their powerful feature-extraction ability, have led to the development of deep DA models (Long et al., 2015; Mingsheng Long and Wang Jianmin, 2016; Chen et al., 2019; Lee et al., 2019; Ding et al., 2018; Tang and Jia, 2019). Contemporary affective models often use deep transfer learning methods like domain-adversarial neural networks (DANN) (Ganin et al., 2016). Although these models can reduce domain distribution differences in large datasets, they have not fully resolved the domain-shift problem with small datasets(i.e., EEG datasets).

In this study, we propose a novel Domain Adaptive Deep Possibilistic clustering (DADPc) approach for EEG-based emotion recognition. It integrates an adaptive loss function with a fuzzy entropy regularization mechanism to enhance the model's cross-domain adaptability and clustering performance. The main contributions are as follows:

- Integrating clustering with neural network training, creating a DADPc criterion for simultaneous feature reconstruction and clustering based on deep features.
- Using a robust loss function with adaptive weights and fuzzy entropy increases insensitivity to outliers and introduces fuzzy entropy regularization for the affinity matrix. The affinity and possibilistic centroid matrices are updated efficiently without using stochastic gradient descent.
- Demonstrating the effectiveness of the proposed method through extensive experiments on multiple EEG datasets (SEED, SEED-IV, and DEAP).

## 2 Related research

Over the past decade, research on using EEG signals for emotion recognition has burgeoned (Shi et al., 2013; Zheng and Lu, 2015; Li et al., 2016; Alarco and Fonseca, 2019; Luo et al., 2024; Zhong et al., 2020; Chen et al., 2021; Zheng and Lu, 2013). Early on, Shi et al. (2013) employed EEG features and SVMs for emotion classification. With advancements in deep learning, deep neural networks gained popularity in EEG-based emotion recognition (Luo et al., 2024; Zhou et al., 2023; Zhong et al., 2020). These models excel in subject-dependent tasks but struggle with subject-independent tasks due to inter-subject EEG variability (Luo et al., 2024; Zhou et al., 2023; Zheng and Lu, 2013).

To address this issue, transfer learning strategies have been implemented. Zheng and Lu (2013) utilized non-deep transfer learning methods such as TCA (Pan et al., 2011) and TPT for cross-subject emotion recognition. Jin et al. (2017) introduced DANN-based deep transfer learning for EEG emotion recognition, outperforming non-deep methods. Following this development, refined DANN-based architectures have emerged (Li et al., 2019b; Peng et al., 2022). For example, Li et al. (2019b) minimized distribution divergence, while Peng et al. (2022) proposed JAGP, both of which enhance cross-subject performance. However, deep transfer learning has limitations (Luo et al., 2024) regarding label noise or small datasets.

Given these challenges, new transfer learning techniques and shallow methods are needed, especially for using labeled source data alongside unlabeled data augmented by pseudo-labels (Peng et al., 2023, 2022; Magdiel and Gibran, 2023; Zhou et al., 2023; Tao and Dan, 2021; Tao et al., 2023; Dan et al., 2022). Zhang and Etemad (2022) introduced the PARSE model to address domain distribution mismatch in semi-supervised EEG emotion recognition, enhancing cross-subject performance. Dan et al. (2024) proposed a semi-supervised model with possibilistic clustering, which requires less labeled data.

To improve the accuracy of shallow methods, we explore a possibilistic clustering method with deep learning features to develop a classifier. Our aim is to address the challenges posed by noise and the limitations of small-scale datasets while enhancing the accuracy of emotion recognition.

## 3 Preliminary

In this study, matrices are denoted by uppercase bold letters, while vectors are represented by lowercase bold letters. For a given matrix **Q**, **q**^*i*^ refers to the *i*^th^ row vector, **q**_*i*_ represents the *i*^th^ column vector, and *q*_*ij*_ denotes the element at position (*i, j*). Additionally, **Q**^*T*^ signifies the transpose of **Q**. The vector **1** = [1, 1, ⋯ , 1]^*T*^ consists entirely of ones, while **0** denotes a zero matrix. The notation **Q**>0 indicates that all elements of the matrix are positive. The ℓ_1_-norm and ℓ_2_-norm of a vector **q** are expressed as ||**q**||_1_ and ||**q**||_2_, respectively. For a scalar-output function *f*(**x**), the gradient is given by $\nabla_{\text{x}}f\left(\text{x}\right)={\left[\frac{\partial f}{\partial x_{1}},\frac{\partial f}{\partial x_{2}},\cdots \;,\frac{\partial f}{\partial x_{n}}\right]}^{T}$. For a vector-output function ***g***(*x*), the gradient with respect to *x* is denoted as $\nabla_{x}g\left(x\right)=\left[\frac{\partial g_{1}}{\partial x},\frac{\partial g_{2}}{\partial x},\cdots \;,\frac{\partial g_{n}}{\partial x}\right]$.

In DA learning, the source domain is defined as $D_{S}={\left\{x_{i}^{s},y_{i}^{s}\right\}}_{i=1}^{n_{s}}$, where the sample set is defined as $X^{s}=\left[x_{1}^{s},\ldots ,x_{n_{s}}^{s}\right]\in {\mathbb{R}}^{d\times n_{s}}$, and the corresponding class labels are defined as $Y^{s}={\left\{y_{1},\ldots ,{\text{y}}_{n_{s}}\right\}}^{T}\in {\left\{0,1\right\}}^{n_{s}\times K}$. *d* is the sample(i.e., *x*_*i*_) dimension of the source domain. *n*_*s*_ is the sample number of the source domain. *K* is the class number of the source domain. Here, $y_{i}\in {\left\{0,1\right\}}^{K\times 1}$ is a one-hot encoded vector; if *x*_*i*_ belongs to the *j*-th class, then *y*_*ij*_ = 1, the rest of the elements of *y*_*i*_ are 0. The unlabeled target domain is defined as $D_{T}={\left\{x_{j}^{t}\right\}}_{j=1}^{n_{t}}$, where the sample set and unknown sample labels during training are $X^{t}=\left[x_{1}^{t},\ldots ,x_{n_{t}}^{t}\right]\in {\mathbb{R}}^{d\times n_{t}},Y^{t}={\left\{y_{1},\ldots ,y_{n_{t}}\right\}}^{T}\in {\mathbb{R}}^{n_{t}\times K},\;$ respectively. *n*_*t*_ is the sample number of target domain. We further define *X* = [*X*^*s*^, *X*^*t*^]∈ℝ^*d*×*N*^ and *Y* = [*Y*^*s*^, *Y*^*t*^]∈ℝ^*N*×*K*^, *N* = *n*_*s*_+*n*_*t*_.

### 3.1 Adaptive loss function

For any given vector **q**, the ℓ_1_-norm and the squared ℓ_2_-norm are defined as $\left||\text{q}|{|}_{1}=\underset{i}{\sum }|q_{i}\right|$ and $||\text{q}|{|}_{2}^{2}=\underset{i}{\sum }q_{i}^{2}$, respectively. To leverage the benefits of different norms, a robust loss function, known as the adaptive loss function, is defined as Nie et al. (2013):


(1) $$\begin{array}{l} ||\text{Q}|{|}_{\sigma }=\underset{i}{\sum }\frac{\left(1+\sigma \right)||{\text{q}}^{i}|{|}_{2}^{2}}{||{\text{q}}^{i}|{|}_{2}+\sigma } \end{array}$$

, where σ serves as a trade-off parameter that governs robustness to different types of outliers. The properties of ||**Q**||_σ_ are summarized in Nie et al. (2013).

### 3.2 Possibilistic clustering

In a specific reproducing kernel Hilbert space (RKHS), denoted as H, the data from the original space can undergo a non-linear transformation ϕ that maps it into a feature representation within the RKHS (Mingsheng Long and Wang Jianmin, 2016). This transformation is of the form $\phi :{\mathbb{R}}^{d}\to H$. The associated kernel function, designated as *K*(..):*X*×*X* → ℝ, is defined by $K\left(x_{1},x_{2}\right)={\langle \phi \left(x_{1}\right),\phi \left(x_{2}\right)\rangle }_{H}$ for *x*_1_, *x*_2_∈*X*. This kernel technique is widely utilized in contemporary non-linear learning approaches (Pan et al., 2011; Long et al., 2015). Research has demonstrated (Gretton et al., 2009; Bruzzone and Marconcini, 2010) that mapping sample data to a high- or infinite-dimensional space can facilitate the capture of higher-dimensional data features (Carlucci et al., 2017). In an RKHS, the maximum mean discrepancy (MMD) criterion effectively gauges the distance between two distributions. Accordingly, let F represent a set of functions of a particular kind, f:X→ℝ. The MMD between two domain distributions, ℙ and ℚ, is defined as follows:


(2) $$\begin{array}{l} MMD_{F}\left[\mathbb{P},\mathbb{Q}\right]:=\underset{f\in F}{\sup}\left(\underset{\mathbb{P}}{\text{E}}\left[f\left(x\right)\right]-\underset{\mathbb{Q}}{\text{E}}\left[f\left(x\right)\right]\right). \end{array}$$

The maximum mean discrepancy (MMD) metric aims to reduce the anticipated disparity between two domain distributions using a specific function *f*, thereby maximizing their similarity. As the size of the domain sample becomes sufficiently large (or approaches infinity), the anticipated disparity converges to (or matches) the empirical mean difference. Consequently, Equation 3 can be expressed in terms of the empirical MMD form.


(3) $$\begin{array}{l} MMD\left(X^{s},X^{t}\right):=\overset{n_{s}}{\underset{i=1}{\sum }}\overset{n_{t}}{\underset{j=1}{\sum }}\|\frac{1}{n_{s}}\overset{n_{s}}{\underset{i=1}{\sum }}\phi \left(x_{i}^{s}\right)-\frac{1}{n_{t}}\overset{n_{t}}{\underset{j=1}{\sum }}\phi \left(x_{j}^{t}\right){\|}_{M}^{2}. \end{array}$$

To establish the universal link between the conventional Maximum Mean Discrepancy (MMD) criterion and the clustering model based on means, we present the theorem below:

**Theorem 1**. The Maximum Mean Discrepancy (MMD) metric can be framed approximately as a unique type of clustering task with a single cluster centroid, denoted by μ, where the assignment of instances to the cluster is represented by ζ_*k*_.


(4) $$\begin{array}{l} MMD\left(X^{s},X^{t}\right)\leq \overset{N}{\underset{k=1}{\sum }}\zeta_{k}\|\phi \left(x_{k}\right)-\mu {\|}_{H}^{2} \end{array}$$

**Proof**.


(5) $$\begin{array}{l} MMD\left(X^{s},X^{t}\right)\\ =\|\frac{1}{n_{s}}\overset{n_{s}}{\underset{i=1}{\sum }}x_{i}^{s}-\frac{1}{n_{t}}\overset{n_{t}}{\underset{j=1}{\sum }}x_{j}^{t}{\|}_{H}^{2}\\ =\|\frac{1}{n_{s}}\overset{n_{s}}{\underset{i=1}{\sum }}x_{i}^{s}-\mu +\mu -\frac{1}{n_{t}}\overset{n_{t}}{\underset{j=1}{\sum }}x_{j}^{t}{\|}_{H}^{2}\\ \leq \|\frac{1}{n_{s}}\overset{n_{s}}{\underset{i=1}{\sum }}x_{i}^{s}-\mu {\|}_{H}^{2}+\|\frac{1}{n_{t}}\overset{n_{t}}{\underset{j=1}{\sum }}x_{j}^{t}-\mu {\|}_{H}^{2}\\ =\frac{1}{n_{s}^{2}}\|\overset{n_{s}}{\underset{i=1}{\sum }}x_{i}^{s}-n_{s}\mu {\|}_{H}^{2}+\frac{1}{n_{t}^{2}}\|\overset{n_{t}}{\underset{j=1}{\sum }}x_{j}^{t}-n_{t}\mu {\|}_{H}^{2}\\ =\frac{1}{n_{s}^{2}}\|\overset{n_{s}}{\underset{i=1}{\sum }}\left(x_{i}^{s}-\mu \right){\|}_{H}^{2}+\frac{1}{n_{t}^{2}}\|\overset{n_{t}}{\underset{j=1}{\sum }}\left(x_{j}^{t}-\mu \right){\|}_{H}^{2}\\ \leq \frac{1}{n^{2}}\overset{n_{s}}{\underset{i=1}{\sum }}\|x_{i}^{s}-\mu {\|}_{H}^{2}+\frac{1}{n_{t}^{2}}\overset{n_{t}}{\underset{j=1}{\sum }}\|x_{j}^{t}-\mu {\|}_{H}^{2}\\ =\overset{N}{\underset{k=1}{\sum }}\zeta_{k}\|x_{k}-\mu {\|}_{H}^{2}, \end{array}$$

where *μ* = *δμ* + (1 − *δ*)*μ*_*t*_ is the cluster center with 0 ≤ δ ≤ 1. μ_*s*_ and μ_*t*_ are the means of the source domain and the target domain, respectively. When *n*_*s*_ = *n*_*t*_, let δ = 0.5. When *n*_*s*_≠*n*_*t*_, the number of data in the source domain and target domain can be set the same during sampling. The sample membership ζ_*k*_ is defined as follows:


(6) $$\zeta_{k}=\{\begin{array}{ll} \frac{1}{n_{s}^{2}}, & x_{k}\in X^{s}\\ \frac{1}{n_{t}^{2}}, & x_{k}\in X^{t} \end{array}$$

Theorem 1 highlights the intrinsic connection between the MMD criterion for domain distribution and the clustering model. This connection can facilitate more efficient alignment of distributions across distinct domains through the clustering of domain data. However, it is important to note that traditional clustering models are susceptible to noise, as pointed out by by Dan et al. (2024). Consequently, DA methods relying on MMD often face the challenge of domain mean shift due to noisy data. To tackle this problem, this paper explores more robust clustering approaches and introduces a novel, effective criterion for measuring domain distribution distance in the following section.

## 4 Methodology

**Figure 1:**
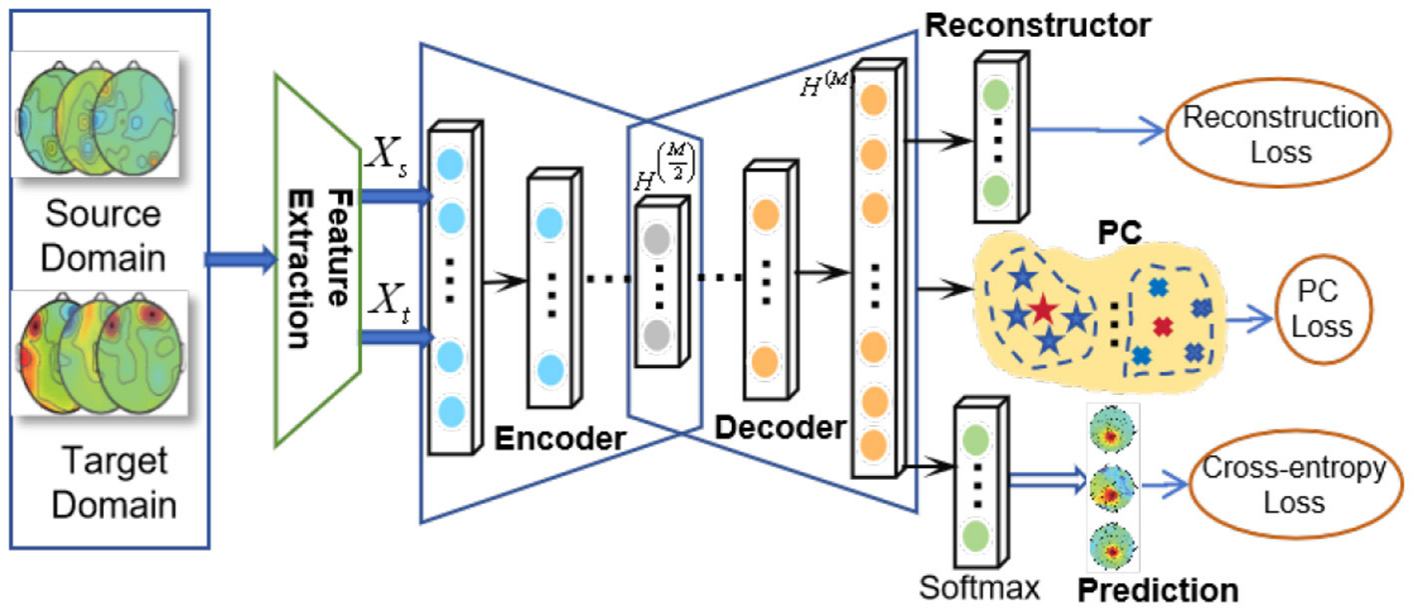
The architecture of DADPc: X_s_ and X_t_ [i.e., H^(0)^] are inputs of the auto-encoder, H^(*M*)^ is the output of the auto-encoder, i.e., the reconstruction of raw data, and ${\text{H}}^{\left(\frac{M}{2}\right)}$ represents the deep features.

Traditional methods such as Kernel K-Means (KKM) and Possibilistic clustering are impractical for large-scale datasets, while efficient algorithms like K-means and Possibilistic clustering are overly simplistic for non-linear data. To address this issue, we propose the DADPc model, which performs effectively on both large datasets and non-linearly distributed data. This section begins by introducing the adaptive loss function and entropy regularization to enhance possibilistic clustering. The architecture of our proposed method is depicted in Figure 1. Our method efficiently utilizes concurrent deep features from the source and target domains for domain adaptation, based on the general framework outlined in Section 4.1. In particular, we enhance the sample reconstruction process using an encoder-decoder deep neural network, as described in Section 4.2, and present our technique for generating the source domain with an adaptive loss function in Section 4.3. Additionally, we introduce a deep clustering approach that incorporates a memory bank and possibilistic theory in Section 4.4. This approach enables us to efficiently extract the essential features from both domains while guaranteeing the cross-domain transferability of the acquired features. Subsequently, the specifics of the DADPc model are discussed in the following subsections.

### 4.1 General formulation

For the problem of DA in complex structures and noisy environments, we aim to improve the robustness of distribution distance metrics for DA and enhance generalization in the target domain. Based on the DA generalization error theory (Ben-David et al., 2010), we seek to achieve the following two core objectives: First, we construct a robust distribution distance metric that can resist the impact of noise, addressing the issue of domain mean-shift. The differences in domain distribution can be selectively corrected. Second, we effectively perform semantic reasoning in the target domain by maintaining the geometric structure consistency of the data in the domain, connecting the discriminative information of the source domain, and minimizing the discriminative error in the target domain. A highly generalizable target domain classifier is constructed. Therefore, our general framework can be described as follows:


(7) $$\begin{array}{l} \underset{\Gamma }{\min}J=\underset{{\text{W}}^{\left(m\right)},{\text{b}}^{\left(m\right)},\text{V},\text{C}}{\min}J_{1}+J_{2}+J_{3} \end{array}$$

, where Γ = {**W**^(*m*)^, **b**^(*m*)^, **V**, **C**}, $J_{1}$, $J_{2}$ and $J_{3}$ are designed for different purposes. $J_{1}$ ensures the minimum reconstruction error based on data from both domains. $J_{2}$ is the classification error for the source domain and a regularization used to avoid the overfitting of auto-encoders, and also able to prevent the auto-encoder from generating a trivial map. $J_{3}$ is the cost function of deep possibilistic clustering with a memory bank. Inspired by the adaptive loss function outlined in Equation 1, which serves as an interpolation between the ℓ_2, 1_-norm and the Frobenius norm squared, we introduce a new objective function for feature reconstruction, the parameters (i.e., *W* and *b*) of deep neural networks, and deep possibilistic clustering with an adaptive loss in $J_{1}$, $J_{2}$ and $J_{3}$.

### 4.2 Feature reconstruction

The DADPc employs a neural network architecture comprising (*M*+1) layers to transform raw data into a nonlinear feature space, where M is an even integer. The initial $\frac{M}{2}$ hidden layers function as an encoder, responsible for reducing the dimensionality of the input data. Conversely, the remaining $\frac{M}{2}$ hidden layers act as a decoder, tasked with the reconstruction of the data. Given that **H**^(0)^ = **X** ∈ ^*d* × *N*^, where **X** belongs to the union of source and target datasets *X*_*s*_∪*X*_*t*_, and *N* equals the sum *n*_*s*_+*n*_*t*_ representing the data counts in each domain, ${\text{h}}_{i}^{\left(0\right)}$ signifies the *i*-th column vector of **H**^(0)^, equivalent to **x**_*i*_ and **x**_*i*_∈**X**. The output generated by the *m*-th layer is denoted as follows:


(8) $$\begin{array}{l} {\text{h}}_{i}^{\left(m\right)}=f\left({\text{W}}^{\left(m\right)}{\text{h}}_{i}^{\left(m-1\right)}+{\text{b}}^{\left(m\right)}\right)\in {\mathbb{R}}^{d_{m}} \end{array}$$

where *m* ranges from 1 to *M*, *d*_*m*_ represents the number of neurons in the *m*-th layer. The activation function for each layer is denoted by *f*(·). The weight matrix and bias for the respective layer, as indicated in Equation 8, are given by **W**^(*m*)^ and **b**^(*m*)^. When presented with a data point **x**_*i*_, the auto-encoder network initially maps the raw data onto a non-linear, low-dimensional space, which can be expressed as follows:


(9) $$\begin{array}{l} {\text{H}}^{\left(\frac{M}{2}\right)}=\left[{\text{h}}_{1}^{\left(\frac{M}{2}\right)},{\text{h}}_{2}^{\left(\frac{M}{2}\right)},\cdots \;,{\text{h}}_{n}^{\left(\frac{M}{2}\right)}\right]\in {\mathbb{R}}^{d_{\frac{M}{2}}\times N} \end{array}$$

then reconstructs the feature as:


(10) $$\begin{array}{l} {\text{H}}^{\left(M\right)}=\left[{\text{h}}_{1}^{\left(M\right)},{\text{h}}_{2}^{\left(M\right)},\cdots \;,{\text{h}}_{n}^{\left(M\right)}\right]\in {\mathbb{R}}^{d\times N} \end{array}$$

We employ deep neural networks to reconstruct the features from the source domain and the target domain, which encompass deep learning, meticulous analysis, and the effective transformation of both domain features. It aims to generate new features that are highly similar to the original or possess specific attributes.


(11) $$\begin{array}{l} J_{1}=\min_{{\text{W}}^{\left(m\right)},{\text{b}}^{\left(m\right)}}\|{\text{H}}^{\left(M\right)}-\text{X}{\|}_{\sigma }^{2}, \end{array}$$

, where **X** = ∪_*k* = 1...*K*_**X**_*c*_*k*__, **X**_*c*_*k*__ includes all *x*_*i*_ which belong to class *k*, and ${\text{x}}_{i}\in {\mathbb{R}}^{d\times 1}$.

By leveraging data reconstruction techniques, the model captures the intrinsic characteristics and structural information of the data, thereby extracting more expressive features while filtering out noise and optimizing data quality. This series of operations enhances the robustness and generalization capabilities of subsequent classification and clustering tasks.

### 4.3 Source classifier

To learn a deep source classification model $h_{s}:X_{s}\to Y_{s}$, we aim to minimize the cross-entropy loss $J_{2}$


(12) $$\begin{array}{l} J_{2}=-\overset{n_{s}}{\underset{i=1}{\sum }}\overset{K}{\underset{k=1}{\sum }}q_{k}\log p_{k}\left(x_{i}^{s}\right)+\lambda_{1}\overset{M}{\underset{m=1}{\sum }}\|{\text{W}}^{\left(m\right)}{\|}_{\sigma }^{2}\\ +\lambda_{2}\overset{M}{\underset{m=1}{\sum }}\|{\text{b}}^{\left(m\right)}{\|}_{\sigma 1}^{2}, \end{array}$$

where ${\text{x}}_{i}\in {\text{X}}_{c_{k}}^{s}$, *q* denotes a one-hot encoding of $y_{i}^{s}$ where *q*_*k*_ is “1” for the correct class and “0” for the rest. The last two terms are regularization used to avoid the overfitting of auto-encoders with regularization parameters λ_1_ and λ_2_. The two terms also prevent the auto-encoder from generating a trivial map. $p_{k}\left(x_{i}\right)=\frac{\exp\left(h_{ik}^{\left(M\right)}\right)}{\overset{K}{\underset{j=1}{\sum }}\exp\left(h_{ij}^{\left(M\right)}\right)}$ represents the predicted probability that sample *x*_*i*_ belongs to class *k*. Before applying the cross-entropy loss to the predictions of the source domain classifier, we first use the sharpening technique to address the ambiguity in the predictions of the source domain data:


(13) $$\begin{array}{l} {\tilde{p}}_{k}\left(x_{i}^{s}\right)=p_{k}{\left(x_{i}^{s}\right)}^{-\tau }/\overset{K}{\underset{e=1}{\sum }}p_{e}{\left(x_{i}^{s}\right)}^{-\tau }. \end{array}$$

where τ represents the temperature parameter utilized for scaling prediction probabilities. When τ approaches 0, the probability distribution converges to a single point mass (Lee, 2013). Thus, Equation 12 can be reformulated as follows:


(14) $$\begin{array}{l} J_{2}=-\overset{n_{s}}{\underset{i=1}{\sum }}\overset{K}{\underset{k=1}{\sum }}q_{k}\log{\tilde{p}}_{k}\left(x_{i}^{s}\right)+\lambda_{1}\overset{M}{\underset{m=1}{\sum }}\|{\text{W}}^{\left(m\right)}{\|}_{\sigma }^{2}\\ +\lambda_{2}\overset{M}{\underset{m=1}{\sum }}\|{\text{b}}^{\left(m\right)}{\|}_{\sigma_{1}}^{2}, \end{array}$$

### 4.4 Deep possibilistic clustering with memory Bank

Recent studies have shown the efficacy of probabilistic clustering methods in mitigating the adverse effects of noise on clustering outcomes (Dan et al., 2021). Consequently, this section generalizes the initial one-cluster center method (Theorem 1) to the context of deep probabilistic one-cluster centering. Subsequently, we introduce a distance metric for deep possibilistic clustering distributions, termed DPC. By incorporating the concept of deep possibilistic clustering entropy, we extend the rigid clustering method of MMD to a more flexible clustering framework. In this framework, the contribution of each sample is weighted based on its proximity to the overall domain mean: data farther from the mean contribute less and are more likely to be viewed as noise. Thus, DPC modulates the influence of noise-induced mean shift during domain alignment. The formula for the deep possibilistic clustering distribution distance metric at the *M*^*th*^ layer is defined as follows:


(15) $$\begin{array}{l} J_{3}=\overset{N}{\underset{i=1}{\sum }}\overset{K}{\underset{k=1}{\sum }}v_{i,k}^{2}\|{\text{h}}_{i}^{\left(M\right)}-{\text{c}}_{k}{\|}_{\sigma } \end{array}$$

The *k*-th cluster centroid is denoted by ${\text{c}}_{k}\in {\mathbb{R}}^{d\times 1}$. The initial cluster centroids are derived from the source domain, as it provides labels for each sample. The cluster centroid for the emotion category *k* can be calculated by averaging all sample features that belong to this category, expressed as follows:


(16) $$\begin{array}{l} c_{k}=\frac{1}{\left|X_{c_{k}}^{s}\right|}\underset{x_{i}^{s}\in X_{c_{k}}^{s}}{\sum }h_{i}^{\left(M\right)} \end{array}$$

*v*_*i, k*_ represents the possibility membership of the feature $h_{i}^{\left(M\right)}$, extracted from the sample *x*_*i*_ in the M layer of the Encoder-Decoder, belonging to the *k*-th class.

To enhance the resilience and efficacy of the possibilistic clustering method for measuring distribution distance in noisy datasets, a regularization term involving fuzzy entropy is introduced in Equation 17. This term is associated with the parameter *v*_*i, k*_:


(17) $$\begin{array}{l} J_{3}=\lambda_{3}\overset{N}{\underset{i=1}{\sum }}\overset{K}{\underset{k=1}{\sum }}\left(v_{ik}^{2}\|{\text{h}}_{i}^{\left(M\right)}-{\text{c}}_{k}{\|}_{\sigma }+P_{e}\left(v_{ik}\right)\right) \end{array}$$

, where $P_{e}\left(v_{ik}\right)=v_{ik}^{2}\ln\;v_{ik}^{2}-v_{ik}^{2}$, λ_3_ acts as a tunable balancing coefficient, ensuring that the values of pertinent data *v*_*ik*_ stay elevated, thus preventing the derivation of non-discriminatory, trivial solutions. The enhanced DADPc model now exhibits a monotonic decrease as *v*_*ik*_ diminishes. This model utilizes the fuzzy entropy term [i.e., *P*_*e*_(*v*_*ik*_)] in Equation 17 to mitigate the adverse effects of noisy data on classification outcomes. The greater the fuzzy entropy is, the more the discriminative information amount of the samples increases, which plays a positive role in enhancing the robust effectiveness of the distribution distance metric. Moreover, fuzzy entropy can effectively limit the influence of noisy or abnormal data in domain distribution alignment. For a comprehensive discussion and empirical insights into how fuzzy entropy enhances robustness, see the analysis in reference (Gretton et al., 2009).

Since each batch of training data includes both the source and target domains in DPC, and since *c*_*k*_ is initialized solely with data from the source domain, it cannot update *c*_*k*_ in real-time during the training stage. To address this issue, we employ a memory bank strategy. The Memory Bank is designed to preserve cluster centroids and their corresponding feature vectors from both domains, which are mapped according to their respective data clusters. We apply the *L*_2_ -norm technique to normalize the feature vectors $h_{i}^{\left(M\right)}$, resulting in normalized features denoted as $\|h_{i}^{\left(M\right)}{\|}_{2}$ alongside the cluster centroids. These values are updated through an iterative process. To estimate real-time probabilities for generating pseudo-labels, the memory bank stores the cluster centroids *c*_*k*_. Additionally, the feature vectors $h_{i}^{\left(M\right)}$ stored in the memory bank are utilized to compute the latest cluster centroids and update the outdated *c*_*k*_ in the memory bank after each training epoch at the final decoder layer. The initial cluster centroids stored in the memory bank originate from the source domain.

Let ***B***∈*R*^(*N*+*K*) × *d*^ be a memory bank that retains the features of all data from both the source and target domains, along with the cluster centroids. Here, *d* signifies the dimensionality of the features in the final linear layer.


(18) $$\begin{array}{l} B=\left[h_{1}^{\left(M\right)},h_{2}^{\left(M\right)},\ldots h_{N}^{\left(M\right)},{\text{c}}_{1},{\text{c}}_{2},\ldots ,{\text{c}}_{K}\right], \end{array}$$

where $h_{i}^{\left(M\right)}$ and *c* undergo *L*_2_ normalization. To account for samples not present in the current mini-batch, we utilize a memory bank to store features and compute similarities, following the approach outlined in Saito et al. (2020). During each iteration, the memory bank ***B*** is updated with features from the mini-batch. Let $h_{i}^{\left(M\right)}$ represent the features within the mini-batch, and let *T*_*b*_ denote the set of indices corresponding to the samples in the mini-batch. For every *i* in *T*_*b*_, we establish:


(19) $$\begin{array}{l} B_{i}=h_{i}^{\left(M\right)}. \end{array}$$

As a result, the memory bank ***B*** comprises the recently updated features from the current mini-batch, the older features that are not included in the mini-batch, and the *K* cluster centroids. Unlike Saito et al. (2020), our approach to updating the memory involves storing features directly, without considering the momentum of features from prior epochs.

### 4.5 Final formulation

The DADPc model is proposed by embedding the objective function of possibilistic clustering with entropy regularization and adaptive loss defined in Equation 20 using an auto-encoder network as


(20) $$\begin{array}{l} \Theta ({\text{W}}^{\left(m\right)},{\text{b}}^{\left(m\right)},\text{V},\text{C})=\min_{{\text{W}}^{\left(m\right)},{\text{b}}^{\left(m\right)},\text{V},\text{C}}{\left\|{\text{H}}^{\left(M\right)}-\text{X}\right\|}_{\sigma }^{2}\\ -\sum_{i=1}^{n_{s}}\sum_{k=1}^{K}q_{k}\log{\tilde{p}}_{k}(x_{i}^{s})+\lambda_{1}\sum_{m=1}^{M}{\left\|{\text{W}}^{\left(m\right)}\right\|}_{\sigma }^{2}\\ +\lambda_{2}\sum_{m=1}^{M}{\left\|{\text{b}}^{\left(m\right)}\right\|}_{\sigma_{1}}^{2}+\lambda_{3}\sum_{i=1}^{N}\sum_{k=1}^{K}(v_{ik}^{2}{\left\|{\text{h}}_{i}^{\left(M\right)}-{\text{c}}_{k}\right\|}_{\sigma }\\ +v_{ik}^{2}\ln v_{ik}^{2}-v_{ik}^{2})\\ \text{ s.t. }0\leq v_{ik}\leq 1. \end{array}$$

Accordingly, DADPc aims to project the raw data onto a nonlinear, low-dimensional feature space and to learn a soft clustering membership matrix using the features mapped nonlinearly simultaneously.

## 5 Optimization algorithms

In this section, we initially devise an effective algorithm to address the adaptive loss function ||.||_σ_, which ensures convergence to a local minimum. Subsequently, we introduce an algorithm aimed at solving the loss function associated with DADPc, as outlined in Equation 20.

### 5.1 Optimization for Weighted Adaptive Loss Function

First, we will examine a broader problem of adaptive loss minimization, formulated as follows:


(21) $$\begin{array}{l} \underset{x}{\min}f\left(x\right)+\underset{i}{\sum }\|g_{i}\left(x\right){\|}_{\sigma }, \end{array}$$

where *g*_*i*_(*x*) yields either a vector or a matrix as output. It is evident that problems ||.||_σ_ in Equation 20 constitute specific instances of Equation 21. The Equation 21 can be rewritten as


(22) $$\begin{array}{l} \underset{x}{\min}f\left(x\right)+\underset{i}{\sum }\frac{\left(1+\sigma \right)\|g_{i}\left(\text{x}\right){\|}_{2}^{2}}{\|g_{i}\left(\text{x}\right){\|}_{2}+\sigma }. \end{array}$$

Building upon the earlier sparse learning optimization algorithms Nie et al. (2013), we introduce an iterative re-weighting approach to address Equation 22. Making the derivative of Equation 22 w.r.t x and equating it to zero, we are able to obtain


(23) $$\begin{array}{l} \nabla f\left(\text{x}\right)+2\left(1+\sigma \right)\underset{i}{\sum }\frac{\|g_{i}\left(\text{x}\right){\|}_{2}+2\sigma }{2{\left(\|g_{i}\left(\text{x}\right){\|}_{2}+\sigma \right)}^{2}}\nabla g_{i}\left(\text{x}\right)\cdot g_{i}\left(\text{x}\right)=0, \end{array}$$

Define


(24) $$\begin{array}{l} d_{i}=\left(1+\sigma \right)\frac{\|g_{i}\left(\text{x}\right){\|}_{2}+2\sigma }{2{\left(\|g_{i}\left(\text{x}\right){\|}_{2}+\sigma \right)}^{2}}, \end{array}$$

Then, Equation 23 can be rewritten as follows:


(25) $$\begin{array}{l} \nabla f\left(\text{x}\right)+2\underset{i}{\sum }d_{i}\nabla g_{i}\left(\text{x}\right)\cdot g_{i}\left(\text{x}\right)=0. \end{array}$$

Note that Equation 25 is still difficult to solve. However, if we fix *d*_*i*_, then Equation 21 is equivalent to


(26) $$\begin{array}{l} \underset{x}{\min}f\left(x\right)+\underset{i}{\sum }d_{i}\|g_{i}\left(\text{x}\right){\|}_{2}^{2}, \end{array}$$

In the given context, the iterative update rule for *d*_*i*_ is defined as $d_{i}\leftarrow \left(1+\sigma \right)\frac{\|g_{i}\left(\text{x}\right){\|}_{2}+2\sigma }{2{\left(\|g_{i}\left(\text{x}\right){\|}_{2}+\sigma \right)}^{2}}$. To address Equation 21, we introduce Algorithm 1. The iterative optimization of the adaptive loss function has been demonstrated to converge in Nie et al. (2013). As Algorithm 1 employs an alternating approach to optimize the adaptive loss, the objective function value decreases monotonically, ensuring the convergence of Algorithm 1.

**Algorithm 1:**
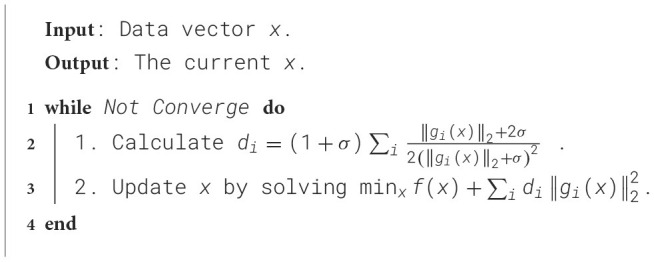
Algorithm to solve Equation 21.

### 5.2 Optimization for DADPc

In this subsection, we will present the details regarding the optimization of Equation 20 using an iterative method known as stochastic gradient descent (SGD). For simplicity, we rewrite Equation 20 as


(27) $$\begin{array}{l} \min_{\Gamma }J=\\ \min_{\Gamma }\frac{1}{2}{\left\|{\text{h}}_{\text{i}}^{\left(M\right)}-{\text{x}}_{\text{i}}\right\|}_{\sigma }^{2}-\sum_{i=1}^{n_{s}}\sum_{k=1}^{K}q_{k}\log{\tilde{p}}_{k}(x_{i}^{s})\\ +\frac{\lambda_{1}}{2}\sum_{m=1}^{M}{\left\|{\text{W}}^{\left(m\right)}\right\|}_{\sigma }^{2}\\ +\frac{\lambda_{2}}{2}\sum_{m=1}^{M}{\left\|{\text{b}}^{\left(m\right)}\right\|}_{\sigma_{1}}^{2}+\lambda_{3}\sum_{i=1}^{N}\sum_{k=1}^{K}(v_{ik}^{2}{\left\|{\text{h}}_{i}^{\left(M\right)}-{\text{c}}_{k}\right\|}_{\sigma }\\ +v_{ik}^{2}\ln v_{ik}^{2}-v_{ik}^{2})\\ \text{ s.t. }0\leq v_{ik}\leq 1 \end{array}$$

where **Γ** = {**W**^(*m*)^, **b**^(*m*)^, **V**, **C**} in order to keep the equations unclustered. As shown in Subsection 5.1, Equation 27 is equivalent to the following dual:


(28) $$\begin{array}{l} \min_{\Gamma }ℒ=\\ \min_{\Gamma }\frac{1}{2}a_{ik}{\left\|{\text{h}}_{\text{i}}^{\left(M\right)}-{\text{x}}_{\text{i}}\right\|}_{2}^{2}-\sum_{i=1}^{n_{s}}\sum_{k=1}^{K}q_{k}\log{\tilde{p}}_{k}(x_{i}^{s})\\ +\frac{\lambda_{1}}{2}\sum_{m=1}^{M}r_{ik}^{w}{\left\|{\text{W}}^{\left(m\right)}\right\|}_{\sigma }^{2}+\frac{\lambda_{2}}{2}\sum_{m=1}^{M}r_{ik}^{b}{\left\|{\text{b}}^{\left(m\right)}\right\|}_{\sigma_{1}}^{2}\\ +\lambda_{3}\sum_{i=1}^{N}\sum_{k=1}^{K}\left(e_{ik}v_{ik}^{2}{\left\|{\text{h}}_{i}^{\left(M\right)}-{\text{c}}_{k}\right\|}_{2}+v_{ik}^{2}\ln v_{ik}^{2}-v_{ik}^{2}\right)\\ \text{ s.t. }0\leq v_{ik}\leq 1 \end{array}$$

where


(29) $$\begin{array}{l} a_{ik}=\left(1+\sigma \right)\frac{\|{\text{h}}_{i}^{\left(M\right)}-{\text{x}}_{i}{\|}_{2}+2\sigma }{2{\left(\|{\text{h}}_{i}^{\left(M\right)}-{\text{c}}_{k}{\|}_{2}+\sigma \right)}^{2}} \end{array}$$

(30) $$\begin{array}{l} r_{ik}^{w}=\left(1+\sigma \right)\frac{\|{\text{W}}^{\left(m\right)}{\|}_{2}+2\sigma }{2{\left(\|{\text{W}}^{\left(m\right)}{\|}_{2}+\sigma \right)}^{2}} \end{array}$$

(31) $$\begin{array}{l} r_{ik}^{b}=\left(1+\sigma_{1}\right)\frac{\|{\text{b}}^{\left(m\right)}{\|}_{2}+2\sigma_{1}}{2{\left(\|{\text{b}}^{\left(m\right)}{\|}_{2}+\sigma_{1}\right)}^{2}} \end{array}$$

(32) $$\begin{array}{l} e_{ik}=\left(1+\sigma \right)\frac{\|{\text{h}}_{i}^{\left(M\right)}-{\text{c}}_{k}{\|}_{2}+2\sigma }{2{\left(\|{\text{h}}_{i}^{\left(M\right)}-{\text{c}}_{k}{\|}_{2}+\sigma \right)}^{2}} \end{array}$$

We can solve Equation 27 by utilizing the coordinate blocking method.

Update **W**^(*m*)^, **b**^(*m*)^ by fixing **V**, **C** : According to the definition of ${\text{h}}_{i}^{\left(m\right)}$ and the back-propagation algorithm, the subgradient of Equation 28 w.r.t. **W**^(*m*)^ and **b**^(*m*)^ can be derived as


(33) $$\{\begin{array}{l} \nabla_{W^{\left(m\right)}}ℒ=\Delta_{i}^{\left(m\right)}h_{i}^{(m-1)T}+\lambda_{1}r_{ik}^{w}W^{\left(m\right)}\\ \nabla_{b^{\left(m\right)}}ℒ=\Delta_{i}^{\left(m\right)}+\lambda_{2}r_{ik}^{b}b^{\left(m\right)} \end{array}$$

where Δ^(*m*)^ is defined as follows:


(34) $$\Delta_{i}^{\left(m\right)}=\{\begin{array}{l} \left(W^{\left(m+1\right)}\Delta_{i}^{\left(m+1\right)}\right)⊙f^{\prime }\left(a_{i}^{\left(m\right)}\right),m\neq M\\ \left(\Theta_{1i}^{\left(M\right)}-\lambda_{1}\Theta_{2i}^{\left(M\right)}+\lambda_{3}\Theta_{3i}^{\left(M\right)}\right)⊙f^{\prime }\left(a_{i}^{\left(M\right)}\right),m=M \end{array}$$

where $\Theta_{1i}^{\left(M\right)}$ and $\Theta_{2i}^{\left(M\right)}$ are defined as


(35) $$\begin{array}{l} \Theta_{1i}^{\left(M\right)}=\left({\text{h}}_{i}^{\left(M\right)}-h_{i}^{\left(0\right)}\right)a_{ik}, \end{array}$$

(36) $$\begin{array}{l} \Theta_{2i}^{\left(M\right)}=\overset{n_{s}}{\underset{i=1}{\sum }}\overset{K}{\underset{k=1}{\sum }}q_{k}\left(1-\frac{\exp\left(h_{ik}^{\left(M\right)}\right)}{\overset{K}{\underset{j=1}{\sum }}\exp\left(h_{ij}^{\left(M\right)}\right)}\right), \end{array}$$

(37) $$\begin{array}{l} \Theta_{3i}^{\left(M\right)}=\left({\text{H}}^{\left(M\right)}-{\text{Y}}_{MB}^{\left(M\right)}{\text{C}}_{MB}^{\left(M\right)}\right){\text{e}}_{ik}{\text{v}}_{ik}^{2} \end{array}$$

⊙ is the element-wise multiplication, *f*′(·) is derivative of the activation function *f*(·), and ${\text{a}}_{i}^{\left(m\right)}$ is the input of m-th layer, i.e., ${\text{a}}_{i}^{\left(m\right)}={\text{W}}^{\left(m\right)}{\text{h}}_{i}^{\left(m-1\right)}+{\text{b}}^{\left(m\right)}$. ${\text{Y}}_{B}^{\left(M\right)}\in {\mathbb{R}}^{N\times K}$. ${\text{C}}_{B}^{\left(M\right)}\in {\mathbb{R}}^{K\times d}$ is from memory bank. According to the cluster centroid $c_{k}\in {\text{C}}_{B}^{\left(M\right)}$, we can obtain the label of each feature vector $y_{i}\in {\text{Y}}_{B}^{\left(M\right)}$ in the *M*-th layer. ${\text{Y}}_{B}^{\left(M\right)}{\text{C}}_{B}^{\left(M\right)}\in {\mathbb{R}}^{N\times d}$. Each row in ${\text{Y}}_{B}^{\left(M\right)}{\text{C}}_{B}^{\left(M\right)}$ corresponds to the cluster centroid of each feature vector.

Based on Equations 33–37 using the SGD algorithm, we update **W**^(*m*)^ and **b**^(*m*)^ as follows:


(38) $$\{\begin{array}{l} {\text{W}}^{\left(m\right)}={\text{W}}^{\left(m\right)}-\mu \nabla_{{\text{W}}^{\left(m\right)}}ℱ\\ {\text{b}}^{\left(m\right)}={\text{b}}^{\left(m\right)}-\mu \nabla_{{\text{b}}^{\left(m\right)}}ℱ \end{array}$$

Using the initial *K* cluster centroids from the source domain, we then acquire new pseudo-labels for the target domain by employing a nearest-centroid classification approach: $y_{t}=\arg\underset{k}{\min}D\left(h_{\left(t,j\right)}^{\left(M\right)},c_{k}\right)$. The distance between *a* and *b* is quantified by *D*(*a, b*). By default, our chosen measure of distance is the cosine similarity metric. Since the feature vectors from $h_{i}^{\left(M\right)}$ and the initial *K* cluster centroids are retained in the memory bank, we update **C** and *y*_*t*_ according to Equation 39 (i.e., ${\text{C}}_{B}^{\left(M\right)}$) after each batch of data training:


(39) $$c_{k}=\frac{\sum_{i=1}^{N}1\left({\hat{y}}_{t}=k\right)h_{i}^{\left(M\right)}}{\sum_{\hat{y}\in Y_{s},{\hat{Y}}_{t}}1\left(\hat{y}=k\right)}$$

Updating **V** by fixing **W**^(*m*)^, **b**^(*m*)^ and **C** through optimizing Equation 20 directly: When **W**^(*m*)^ and **b**^(*m*)^ are fixed, Equation 20 becomes equivalent to:


(40) $$\begin{array}{l} min_{v_{ik}}\overset{N}{\underset{i=1}{\sum }}\overset{K}{\underset{k=1}{\sum }}\lambda_{3}\left(v_{ik}^{2}\|{\text{h}}_{i}^{\left(M\right)}-{\text{c}}_{k}{\|}_{\sigma }+v_{ik}^{2}\ln\;v_{ik}^{2}-v_{ik}^{2}\right),\\ s.t.0\leq v_{ik}\leq 1 \end{array}$$

The Lagrangian function for Equation 39 is represented as


(41) $$\begin{array}{l} min_{v_{ik}}\lambda_{3}\left(\overset{N}{\underset{i=1}{\sum }}\overset{k}{\underset{j=1}{\sum }}v_{ik}^{2}\|{\text{h}}_{i}^{\left(M\right)}-{\text{c}}_{k}{\|}_{\sigma }+v_{ik}^{2}\ln\;v_{ik}^{2}-v_{ik}^{2}\right)\\ -\overset{N}{\underset{i=1}{\sum }}\overset{k}{\underset{j=1}{\sum }}\beta_{ik}\left(v_{ik}\right) \end{array}$$

where β_*ik*_ is aa Lagrangian multiplier with 0 < β_*ik*_ < 1. According to KKT conditions, we have


(42) $$\begin{array}{l} v_{ik}=\exp\left(\frac{1-\lambda_{3}\overset{N}{\underset{i=1}{\sum }}\overset{K}{\underset{k=1}{\sum }}\|{\text{h}}_{i}^{\left(M\right)}-{\text{c}}_{k}{\|}_{\sigma }}{4}\right) \end{array}$$

We can therefore apply an iterative algorithm to update **W**^(*m*)^, **b**^(*m*)^, **V** and **C**. If SGD decreases L in Equation 28, the algorithm will converge to a local minimum, as the optimization can be viewed as a variant of coordinate gradient descent.

The optimization procedure of Equation 27 is summarized in Algorithm 2.

**Algorithm 2:**
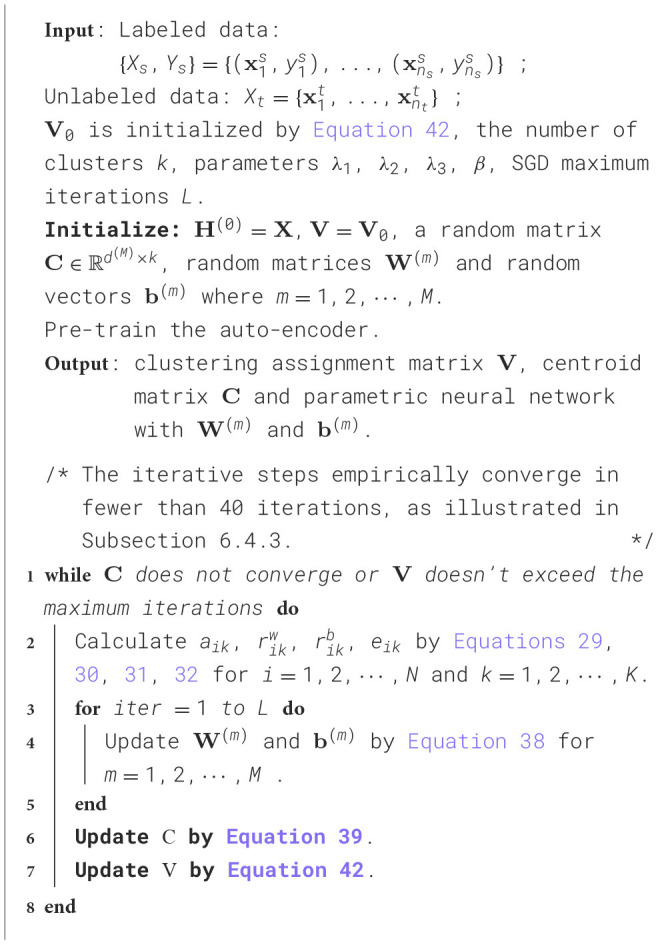
Algorithm to solve Equation 27.

## 6 Experimental analysis

This section employs three well-known benchmark datasets: SEED (Zheng and Lu, 2015), SEED-IV (Zheng and Lu, 2013; Zhou et al., 2023), and DEAP (Sander et al., 2012), to thoroughly assess the model's ability to recognize EEG-based emotions. Specifically, in the following tables of experimental results, the bold values in each table are the best accuracy performance results achieved. Pacc denotes the average accuracy performance of each method.

### 6.1 Datasets

#### 6.1.1 Description

The SEED dataset includes EEG data on emotions collected from 15 individuals who watched 15 movie clips designed to elicit three emotional responses: positive, negative, and neutral. Conversely, the SEED-IV dataset contains EEG emotion data from 15 participants as well but features 24 movie segments aimed at evoking four emotions: happiness, sadness, neutrality, and fear. Both the SEED and SEED-IV datasets involved participants engaging in three sessions, each occurring on separate days with a one-week interval between them. The EEG signals were recorded using a 62-channel ESI Neuroscan system.

Moreover, we are keen to investigate performance in cross-dataset scenarios, specifically exploring whether acceptable recognition precision can be sustained when the training and testing data originate from different subjects, are recorded with varying EEG equipment, and involve emotional states elicited by diverse stimuli. Furthermore, our objective is to assess if multi-source domain adaptation methods can enhance results in such circumstances. To achieve this, we utilize the DEAP dataset (Sander et al., 2012), which is publicly available, to examine emotional states. This dataset includes data from 32 individuals who viewed 40 one-minute music videos intended to evoke emotional reactions, while their physiological responses were recorded. After watching each video, the participants evaluated their emotions in five aspects: valence (how pleasant it was), arousal (the level of excitement), dominance (degree of control), liking (personal preference), and familiarity (recognition of the stimulus). The evaluation scores range from one (the lowest level) to nine (the highest level), but for familiarity, the scores vary from one to five.

To gain a deeper understanding of these three benchmarks, please refer to Lan et al. (2019). As stated in Zhong et al. (2022) and Lan et al. (2019), there are notable differences among these benchmarks. These discrepancies can arise from various factors, including variations in sessions, participants, experimental procedures, EEG equipment, and the types of emotional stimuli used.

#### 6.1.2 Feature extraction

The SEED dataset's EEG data preprocessing followed standard procedures outlined in Luo et al. (2024). First, the EEG signals were downsampled to 200 Hz. Next, artifacts such as electrooculogram (EOG) and electromyography (EMG) were removed. A bandpass filter ranging from 0.3 to 50 Hz was applied to enhance signal quality. Each trial was then divided into several 1-second data segments. The length of each trial in the SEED dataset varied between 185 and 265 seconds, depending on the duration of the emotional stimulus used to evoke the targeted emotion. To ensure consistency across various classes, all trials were shortened to a uniform length of 185s.

The DEAP dataset's EEG data were recorded using Biosemi Active Two devices, initially sampled at 512 Hz and subsequently down-sampled to 128 Hz. In DEAP, emotions are scored on a five-point scale; to align with the SEED dataset, we discretize the emotional dimensions as follows: positive for valence ratings above 7, neutral for ratings between 3 and 7, and negative for ratings below 3. We identify DEAP trials where the majority of participants reported the successful induction of positive, neutral, and negative emotions. Specifically, trial 18 evokes positive emotion, trial 16 evokes neutral emotion, and trial 38 evokes negative emotion, with 27, 28, and 19 participants, respectively, confirming the intended emotion. The participants who consistently reported successful emotion elicitation in these trials (18, 16, and 38) are subjects 2, 5, 10, 11, 12, 13, 14, 15, 19, 22, 24, 26, 28, and 31. Therefore, for the DEAP dataset, we only use the selected trials from these fourteen subjects. Each trial lasts 63 seconds, but the first 3 seconds consist of baseline recordings without emotion elicitation; thus, we use the segment starting from the 4th second, resulting in a 60-second valid trial length.

To capture emotion-related information, we computed differential entropy (DE) features (Zhong et al., 2022; Lan et al., 2019) for five frequency bands: Delta (1–3 Hz), Theta (4–7 Hz), Alpha (8–13 Hz), Beta (14–30 Hz), and Gamma (31–50 Hz). This process generated 310 features (62 channels × 5 frequency bands) for every 1-second data segment, serving as model input. For the DEAP dataset, the ultimate feature vector comprised 160 dimensions (32 channels × 5 frequency bands), with each trial containing 60 samples. In the SEED dataset, the final feature vector had 310 dimensions (62 channels × 5 frequency bands), with each trial yielding 185 samples.

Following the approach in Donahue et al. (2014), our DADPc method can be readily trained utilizing deep features extracted from established models. We fine-tune pre-trained deep models, such as Resnet101 (EEG differential entropy features (62 channels × 5 bands) are reshaped into 62 × 5 matrices, mimicking image input for ResNet-101). The Resnet101's bottleneck layer outputs are used to balance semantic and spatial information for cross-domain alignment. He et al. (2016), on the source domain and then extract deep features from EEG signals. The Resnet101's Bottleneck layer outputs are used to balance semantic and spatial information for cross-domain alignment. Pre-experiments showed ResNet-101's deep residual structure outperforms ResNet-50/DenseNet in cross-domain scenarios, with mature open-source implementations aiding reproducibility.. These deep representations can subsequently be used to train the recognition model.

### 6.2 Experimental protocols

Since the parameter **b** does not require feature selection, the *l*_σ1_-norm on **b** can be fixed as the *l*_2_-norm. To thoroughly assess the robustness and consistency of our proposed DADPc method and facilitate comparison with previous works, we utilize four unique validation protocols that incorporate various evaluation strategies for a comprehensive examination, as outlined in Zhou et al. (2022); Tao et al. (2024):

1) Cross-subject cross-session (CUCE) with leave-one-subject-out (LOSO) cross-validation. To rigorously evaluate the model's durability on novel subjects and sessions, we apply a strict assessment protocol known as CUCE with LOSO. During each cycle, the session data of one subject is designated as the target, while the session data from all other subjects are utilized as the source. This training and validation procedure is repeated until each subject's sessions have served as the target once. Given the inherent variability among individuals and sessions, this evaluation protocol poses a significant challenge for the model's proficiency in EEG-based emotion recognition tasks.
2) Cross-subject single-session (CUSE) with LOSO cross-validation. The most commonly adopted validation technique in EEG-based emotion recognition (Li et al., 2019a; Luo et al., 2018; Li et al., 2019b; Zhou et al., 2022; Luo et al., 2024) involves assigning data from a single session of one subject as the target, while data from all other subjects serve as the source. This iterative training and validation process continues until each subject has been designated as the target once. Consistent with other research, this cross-validation method considers only the first session.
3) Within-subject Cross-session (WUCE) with LOSO cross-validation. Consistent with prevalent techniques, we use a time series cross-validation methodology that leverages historical data to forecast present or future data. In this context, the first two sessions for each subject serve as the source domain, while the following session acts as the target domain. The final results are obtained by calculating the mean accuracy and standard deviation across all subjects.
4) Cross-database cross-validation (CDCV). In line with the configurations specified in Tao and Dan (2021); Tao et al. (2023), we employ 32 common channels from SEED and DEAP to construct a unified feature space of 160 dimensions, enhancing cross-dataset generalization. This enables the formulation of multiple cross-dataset generalization scenarios: DEAP → SI, DEAP → SII, DEAP → SIII, SI → DEAP, SII → DEAP, and SIII → DEAP. Here, “A → B” denotes adapting from dataset A to dataset B, with SI, SII, and SIII denoting Session I, Session II, and Session III of SEED, respectively. When DEAP is the source dataset, 2,520 data points are selected, whereas 2775 data points are chosen from each of the three SEED sessions (SI, SII, and SIII) to serve as the target datasets. Conversely, when each SEED session is the source, we resample 41,625 data points for training and 180 samples from DEAP as the target.

### 6.3 Experimental results

Given that parameter selection remains a pressing issue in machine learning, this study empirically investigates the parameter space to identify the most effective settings. Our approach involves three primary model parameters, initially set to λ_1_, λ_2_, and λ_3_ equal to 0. These parameters are then fine-tuned using cross-validation within the range [10^−4^, 10^5^]. Moreover, for the experimental setup, the matrix norm is set at σ = 0.001. The results are reported as the average performance across all participants.

To compare our method with several recently introduced deep adaptation models, we specifically assess DADPc using deeply extracted features from the neural network with five layers (i.e., 1024-500-300-500-1024). For other deep benchmarks, we directly utilize their publicly available source codes to fine-tune the pre-trained models employed in their respective works.

#### 6.3.1 CUCE with LOSO cross-validation

To assess the effectiveness and consistency of our model across both cross-subject and cross-session contexts, we employ LOSO cross-validation to validate the proposed DADPc approach within the CUCE framework, utilizing the SEED and SEED-IV datasets. The results, presented in Tables 1, 2, reveal that our method outperforms others on both datasets, achieving an emotion recognition accuracy of 87.83 ± 4.21% for three classes on SEED and 75.31 ± 6.22% for four classes on SEED-IV. These findings underscore the superior recognition precision and enhanced generalization capability of the DADPc method, particularly in the presence of complex individual and environmental variations, which suggests improved emotional validity.

**Table 1:** The mean accuracies (%) and standard deviations (%) on the SEED database using CUCE with LOSO cross-validation.

**Methods**	**Pacc**	**Methods**	**Pacc**
**Traditional machine learning methods**			
RF; Breiman (2001)	69.60 ± 7.64	KNN (Duda et al., 2000)	60.66 ± 7.93
SVM; Vapnik (1995)	62.24 ± 5.48	Adaboost (Zhu et al., 2006)	71.87 ± 5.70
TCA; Pan et al. (2011)	65.31 ± 6.04	CORAL (Sun et al., 2015)	69.22 ± 4.11
SA; Fernando et al. (2013)	61.41 ± 9.75	GFK (Gong et al., 2012)	67.36 ± 6.52
DICE; Liang et al. (2019)	73.56 ± 4.23	GAKT (Ding et al., 2018)	74.82 ± 7.14
MDDD; Luo et al. (2024)	76.60 ± 6.79	EDPC	**76.82** **±** **6.14**
**Deep learning methods**			
DCORAL; Sun and Saenko (2016)	80.87 ± 6.04	DAN (Long et al., 2019)	82.51 ± 3.71
DDC; Tzeng et al. (2014)	82.17 ± 4.96	DANN (Ganin et al., 2016)	84.79 ± 6.44
PR-PL; Zhou et al. (2022)	85.56 ± 4.78	PARSE (Zhang and Etemad, 2022)	82.44 ± 5.00
EEGMatch; Zhou et al. (2023)	86.30 ± 5.04	DADPc	**87.83** **±** **4.21**

**Table 2:** The mean accuracies (%) and standard deviations (%) on the SEED-IV database using CUCE with LOSO cross-validation.

**Methods**	**Pacc**	**Methods**	**Pacc**
**Traditional machine learning methods**			
KNN; Duda et al. (2000)	40.83 ± 7.28	SVM (Vapnik, 1995)	51.78 ± 12.85
Adaboost; Zhu et al. (2006)	53.44 ± 9.12	TCA (Pan et al., 2011)	56.56 ± 13.77
CORAL; Sun et al. (2015)	49.44 ± 9.09	SA (Fernando et al., 2013)	64.44 ± 9.46
GFK; Gong et al. (2012)	45.89 ± 8.27	KPCA (Schlkopf et al., 1998)	51.76 ± 12.89
DICE; Liang et al. (2019)	66.75 ± 7.25	GAKT (Ding et al., 2018)	64.48 ± 5.52
MDDD; Luo et al. (2024)	64.90 ± 10.25	EDPC	**67.88** **±** **5.21**
**Deep learning methods**			
DGCNN; Song et al. (2018)	52.82 ± 9.23	DAN (Long et al., 2019)	58.87 ± 8.13
RGNN; Zhong et al. (2020)	73.84 ± 8.02	BiHDM (Li et al., 2019c)	69.03 ± 8.66
BiDANN; Li et al. (2018b)	65.59 ± 10.39	DANN (Ganin et al., 2016)	54.63 ± 8.03
PR-PL; Zhou et al. (2022)	74.92 ± 7.92	PARSE (Zhang and Etemad, 2022)	69.78 ± 8.22
EEGMatch; Zhou et al. (2023)	73.60 ± 7.53	DADPc	**75.31** **±** **6.22**

#### 6.3.2 CUSE with LOSO cross-validation

Tables 3, 4 summarize the experimental outcomes of the LOSO recognition task conducted on the SEED and SEED-IV datasets within the CUSE framework, alongside a benchmarking against previous studies. All results are reported as mean ± standard deviation. Our proposed DADPc model, as shown in these tables, achieves a peak performance of 94.62% with a standard deviation of 4.37%. DADPc outperforms the best-reported literature results by 1.56%, exhibiting a lower standard deviation on the SEED-IV dataset. Notably, the model's performance is superior on the SEED-IV dataset compared to the SEED dataset. This highlights DADPc's efficacy in addressing individual differences and improving robust pseudo-labeling (Litrico et al., 2023) for a wider range of emotion recognition in affective Brain-Computer Interface (aBCI) applications.

**Table 3:** The mean accuracies (%) and standard deviations (%) on the SEED database using CUSE with LOSO cross-validation.

**Methods**	**Pacc**	**Methods**	**Pacc**
**Traditional machine learning methods**			
TKL; Long et al. (2015)	63.54 ± 15.47	T-SVM (Ronan et al., 2006)	68.57 ± 9.54
TCA; Pan et al. (2011)	63.64 ± 14.88	TPT (Zheng and Lu, 2013)	73.86 ± 11.05
KPCA; Schlkopf et al. (1998)	61.28 ± 14.62	GFK (Gong et al., 2012)	71.31 ± 14.09
SA; Fernando et al. (2013)	66.00 ± 10.89	DICA (Ma et al., 2019)	69.40 ± 07.80
DBN; Zheng and Lu (2015)	61.01 ± 12.38	SVM (Vapnik, 1995)	58.18 ± 13.85
DICE; Liang et al. (2019)	74.22 ± 7.33	GAKT (Ding et al., 2018)	72.29 ± 4.66
MDDD; Luo et al. (2024)	**84.57** **±** **9.49**	EDPC	82.34 ± 4.52
**Deep learning methods**			
DGCNN; Song et al. (2018)	79.95 ± 9.02	DAN (Long et al., 2019)	83.81 ± 8.56
RGNN; Zhong et al. (2020)	85.30 ± 6.72	BiHDM (Li et al., 2019c)	85.40 ± 7.53
WGAN-GP; Luo et al. (2018)	87.10 ± 7.10	MMD (Dino et al., 2013)	80.88 ± 10.10
ATDD-DANN; Du et al. (2022)	90.92 ± 1.05	JDA-Net (Li et al., 2019b)	88.28 ± 11.44
R2G-STNN; Li et al. (2022)	84.16 ± 7.63	SimNet (Pinheiro, 2018)	81.58 ± 5.11
BiDANN; Li et al. (2018b)	83.28 ± 9.60	DResNet (Ma et al., 2019)	85.30 ± 8.00
ADA; Philip et al. (2017)	84.47 ± 10.65	DANN (Ganin et al., 2016)	81.65 ± 9.92
PR-PL; Zhou et al. (2022)	93.06 ± 5.12	PARSE (Zhang and Etemad, 2022)	82.11 ± 5.83
EEGMatch; Zhou et al. (2023)	92.45 ± 6.85	DADPc	**93.58** **±** **6.35**

**Table 4:** The mean accuracies (%) and standard deviations (%) on the SEED-IV database using CUSE with LOSO cross-validation.

**Methods**	**Pacc**	**Methods**	**Pacc**
**Traditional machine learning methods**			
TKL; Long et al. (2015)	63.54 ± 15.47	T-SVM (Ronan et al., 2006)	68.57 ± 9.54
TCA; Pan et al. (2011)	63.64 ± 14.88	TPT (Zheng and Lu, 2013)	73.86 ± 11.05
KPCA; Schlkopf et al. (1998)	61.28 ± 14.62	GFK (Gong et al., 2012)	71.31 ± 14.09
SA; Fernando et al. (2013)	66.00 ± 10.89	DICA (Ma et al., 2019)	69.40 ± 07.80
DBN; Zheng and Lu (2015)	61.01 ± 12.38	SVM (Vapnik, 1995)	58.18 ± 13.85
DICE; Liang et al. (2019)	74.22 ± 7.33	GAKT (Ding et al., 2018)	72.29 ± 4.66
MDDD; Luo et al. (2024)	76.60 ± 6.79	EDPC	**76.82** **±** **7.14**
**Deep learning methods**			
DGCNN; Song et al. (2018)	79.95 ± 9.02	DAN (Long et al., 2019)	83.81 ± 8.56
RGNN; Zhong et al. (2020)	85.30 ± 6.72	BiHDM (Li et al., 2019c)	85.40 ± 7.53
WGAN-GP; Luo et al. (2018)	87.10 ± 7.10	MMD (Dino et al., 2013)	80.88 ± 10.10
ATDD-DANN; Du et al. (2022)	90.92 ± 1.05	JDA-Net (Li et al., 2019b)	88.28 ± 11.44
R2G-STNN; Li et al. (2022)	84.16 ± 7.63	SimNet (Pinheiro, 2018)	81.58 ± 5.11
BiDANN; Li et al. (2018b)	83.28 ± 9.60	DResNet (Ma et al., 2019)	85.30 ± 8.00
ADA; Philip et al. (2017)	84.47 ± 10.65	DANN (Ganin et al., 2016)	81.65 ± 9.92
PR-PL; Zhou et al. (2022)	93.06 ± 5.12	PARSE (Zhang and Etemad, 2022)	82.11 ± 5.83
EEGMatch; Zhou et al. (2023)	92.45 ± 06.85	DADPc	**94.62** **±** **4.37**

#### 6.3.3 WUCE with LOSO cross-validation

The outcomes of the WUCE cross-validation for the SEED dataset are outlined in Table 5, and those for the SEED-IV dataset are presented in Table 6.

**Table 5:** The mean accuracies (%) and standard deviations (%) on the SEED database using WUCE with LOSO cross-validation.

**Methods**	**Pacc**	**Methods**	**Pacc**
**Traditional machine learning methods**			
RF; Breiman (2001)	76.42 ± 11.15	KNN (Duda et al., 2000)	72.96 ± 12.10
TCA; Pan et al. (2011)	77.63 ± 11.49	CORAL (Sun et al., 2015)	82.18 ± 9.81
SA; ;Fernando et al. (2013)	67.79 ± 7.43	GFK (Gong et al., 2012)	79.28 ± 7.44
DICE; Liang et al. (2019)	81.58 ± 7.55	GAKT (Ding et al., 2018)	80.31 ± 6.44
MDDD; Luo et al. (2024)	81.27 ± 5. 47	EDPC	**82.31** **±** **6.44**
**Deep learning methods**			
DAN; Long et al. (2019)	89.16 ± 7.90	SimNet (Pinheiro, 2018)	86.88 ± 7.83
DDC; Tzeng et al. (2014)	91.14 ± 5.61	ADA (Philip et al., 2017)	89.13 ± 7.13
DANN; Ganin et al. (2016)	89.45 ± 6.74	MMD (Dino et al., 2013)	84.38 ± 12.05
JDA-Net; Li et al. (2019b)	91.17 ± 8.11	DCORAL (Sun and Saenko, 2016)	88.67 ± 6.25
PR-PL; Zhou et al. (2022)	93.18 ± 6.55	PARSE (Zhang and Etemad, 2022)	89.85 ± 5.06
EEGMatch; Zhou et al. (2023)	**94.70** **±** **4.10**	DADPc	93.18 ± 5.40

**Table 6:** The mean accuracies (%) and standard deviations (%) on the SEED-IV database using WUCE with LOSO cross-validation.

**Methods**	**Pacc**	**Methods**	**Pacc**
**Traditional machine learning methods**			
RF; Breiman (2001)	60.27 ± 16.36	KNN (Duda et al., 2000)	54.18 ± 16.28
TCA; Pan et al. (2011)	59.49 ± 12.07	CORAL (Sun et al., 2015)	66.88 ± 14.67
SA; Fernando et al. (2013)	56.94 ± 11.45	GFK (Gong et al., 2012)	60.66 ± 10.00
DICE; Liang et al. (2019)	69.68 ± 12.52	GAKT (Ding et al., 2018)	68.77 ± 6.00
MDDD; Luo et al. (2024)	68.81 ± 9.25	EDPC	**71.39** **±** **5.22**
**Deep learning methods**			
DCORAL; Sun and Saenko (2016)	65.10 ± 13.20	DAN (Long et al., 2019)	60.20 ± 10.20
DDC; Tzeng et al. (2014)	68.80 ± 16.60	MEERNet (Chen et al., 2021)	72.10 ± 14.10
PR-PL; Zhou et al. (2022)	74.62 ± 14.15	PARSE (Zhang and Etemad, 2022)	70.24 ± 8.47
EEGMatch; Zhou et al. (2023)	72.91 ± 8.34	DADPc	**75.36** **±** **5.13**

During the experiments on the SEED datasets, EEGMatch emerged as the top-performing method. This can be attributed to the mixup technique, which enriched the data and enhanced model training, albeit at the cost of higher computational expenses due to the increased data volume. Nonetheless, DADPc achieved results that were comparable, closely trailing behind. This underscores DADPc's proficiency in categorizing distinct classes. For the SEED-IV dataset, which involves four-class emotion recognition, DADPc excelled, particularly as the number of categories increased. This highlights DADPc's superior accuracy in recognizing more nuanced emotions and its robust scalability.

#### 6.3.4 CDCV results

In this section, we aim to evaluate the extensive and consistent generalization capability of our proposed DADPc method, particularly in the realm of cross-dataset emotion recognition. Essentially, achieving generalization across datasets presents a more formidable challenge than cross-subject generalization due to the significant differences that exist between datasets.

The experimental outcomes for six tasks, as shown in Table 7, indicate that the performance of all methods when applied across datasets is slightly lower compared to their performance within the same dataset. This finding supports the notion that the distributional differences between two datasets are more pronounced than those between two subjects.

**Table 7:** The mean accuracies (%) using CDCV (SI, Session I; SII, Session II; SIII, Session III).

**Methods**	**DEAP → SI**	**DEAP → SII**	**DEAP → SIII**	**SI → DEAP**	**SII → DEAP**	**SIII → DEAP**
**Non-Deep learning methods**						
SA; Fernando et al. (2013)	56.69	59.33	52.28	55.61	48.90	50.02
TPT; Zheng and Lu (2013)	58.23	60.22	55.39	60.01	51.41	52.23
GAKT; Ding et al. (2018)	60.36	61.33	59.40	59.79	52.49	54.16
MDDD; Luo et al. (2024)	61.29	62.15	61.05	**61.81**	**56.16**	**56.68**
DICE; Liang et al. (2019)	60.68	62.79	60.86	60.49	54.78	55.33
EDPC	**62.17**	**63.36**	**62.08**	60.11	54.89	56.45
**Deep learning methods**						
DDG; Ding et al. (2018)	62.40	64.92	73.92	64.29	54.29	53.33
DDC; Tzeng et al. (2014)	60.89	62.43	69.43	62.16	52.16	50.07
DANN; Ganin et al. (2016)	61.08	62.51	72.51	63.77	53.77	52.62
DSAN; Zhu et al. (2021)	63.28	64.50	74.50	64.58	55.58	54.10
DCORAL; Sun and Saenko (2016)	60.15	60.42	70.42	61.54	52.54	51.00
CAN; Kang et al. (2022)	64.22	65.77	**75.77**	**66.12**	57.12	55.39
DADPc	**65.83**	**66.30**	75.16	65.39	**57.59**	**58.28**

Specifically, our DADPc model demonstrates superior performance compared to other baseline methods in 4 out of the 6 recognition tasks. While CAN (Kang et al., 2022) occasionally achieves the best results in two particular settings, DADPc consistently ranks first in other situations. These results suggest that the combined approach of reconstructing feature learning and possibilistic clustering learning is a more effective strategy.

Finally, as observed from Tables 1–7, the performance of DADPc consistently outperforms that of EDPC, achieving a maximum improvement of nearly 18%. This result suggests that utilizing a simple deep neural network for feature extraction and reconstruction can effectively enhance emotion recognition performance.

### 6.4 Discussion

To thoroughly assess the model's efficacy, we conduct additional evaluations to determine the impact of various configurations within the DADPc framework.

#### 6.4.1 Effect of noisy labels

**Figure 2:**
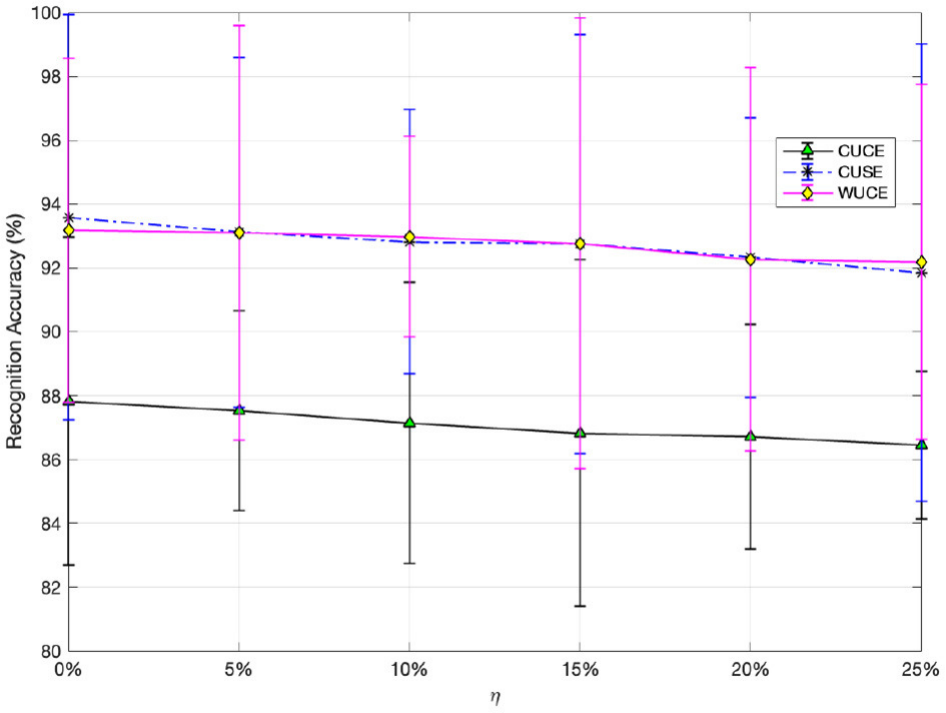
Robustness with different ratios of noise labels.

To evaluate the model's resilience in scenarios with noisy labels, we randomly introduce η% noise to the source labels and assess the model's performance on unseen target data. In particular, we replace η% of the actual labels in *Y*_*s*_ with random labels and then conduct supervised learning. Afterward, we test the trained model on the target domain. It is important to note that noise is only introduced to the source domain, while the target domain is used exclusively for evaluating the model. In our experiments, we vary η% at 5%, 10%, 15%, 20%, and 25%. The model accuracies on the SEED dataset for these noise levels are shown in Figure 2, revealing a minor decline in performance as the label noise ratio rises from 5% to 25%. These results demonstrate that DADPc is a robust model with a high level of tolerance for noisy labels. Building on the study in Zhou et al. (2022), our future research could incorporate the recent method (Jin et al., 2023) to further reduce general noise in EEG signals and improve model stability in cross-subject ER applications.

#### 6.4.2 Visualization and confusion matrix

**Figure 3:**
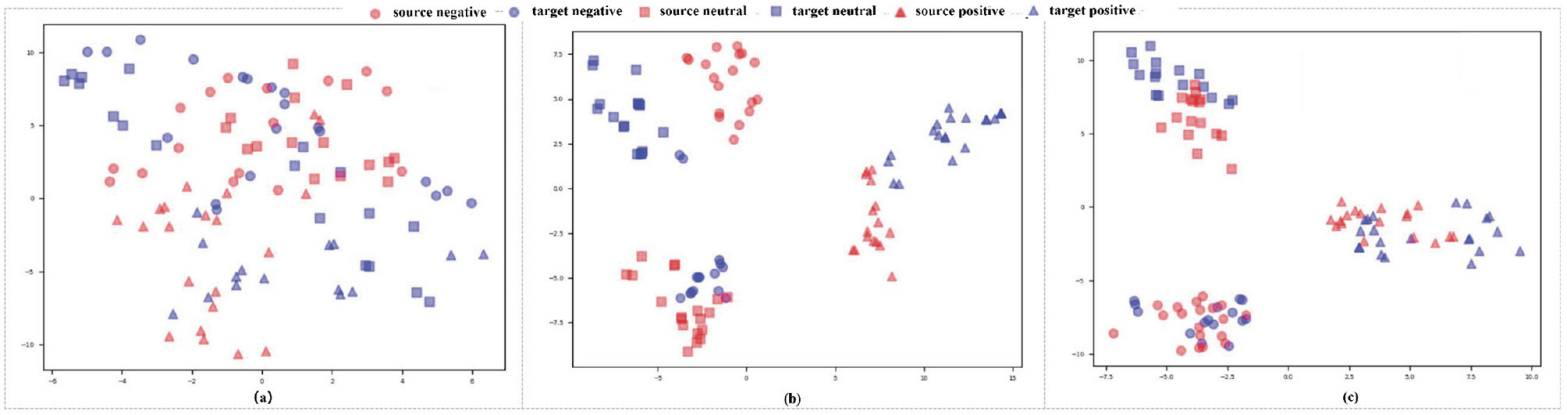
A visualization of the learned feature representations: **(a)** before training, **(b)** at the training epoch of 50, and **(c)** in the final model, respectively.

We use the t-distributed stochastic neighbor embedding (t-SNE) algorithm (Laurens and Hinton, 2008) to visually compare the learning ability of our DADPc at various training stages. The visual results are shown in Figure 3. By comparing Figures 3a–c, we observe:

- The intra-class variation across domains in Figure 3a is larger than in Figures 3b, c. This indicates that deep feature extraction and possibilistic clustering enhance DADPc's efficiency.
- In Figure 3c, feature clusters are denser and less scattered than in Figure 3a. In DADPc, each target feature is drawn to its class-cluster center, while target-cluster centers align with source-cluster centers. This demonstrates that DADPc acquires meaningful features and cluster centers, highlighting its advantage in unsupervised domain adaptation.

**Figure 4:**
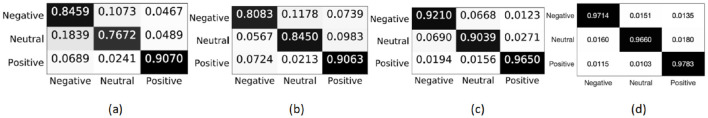
Confusion matrices of different models. **(a)** BiDANN Li et al. (2018b), **(b)** BiHDM Li et al. (2019c), **(c)** PR-PL Zhou et al. (2022), **(d)** DADPc.

To qualitatively assess the model's performance across emotion categories, we visually examined the DADPc model confusion matrix on the SEED dataset using the WUCE metric and compared it with recent models (Li et al., 2018b, 2019c; Zhou et al., 2022). Figure 4 shows that all models distinguish positive emotions well (accuracy exceeding 90%) but struggle to differentiate negative and neutral emotions. BiDANN (Li et al., 2019c) has a recognition rate of less than 80% for neutral emotions (76.72%). Compared to existing methods (Figures 4a–c our model demonstrates better recognition, particularly for neutral-negative emotions. As shown in Figure 4d, our model achieves recognition rates of 97.14%, 96.60%, and 97.83% for negative, neutral, and positive emotions, respectively, outperforming PR-PL and highlighting its adaptability and discriminative power in the target domain.

#### 6.4.3 Convergence

**Figure 5:**
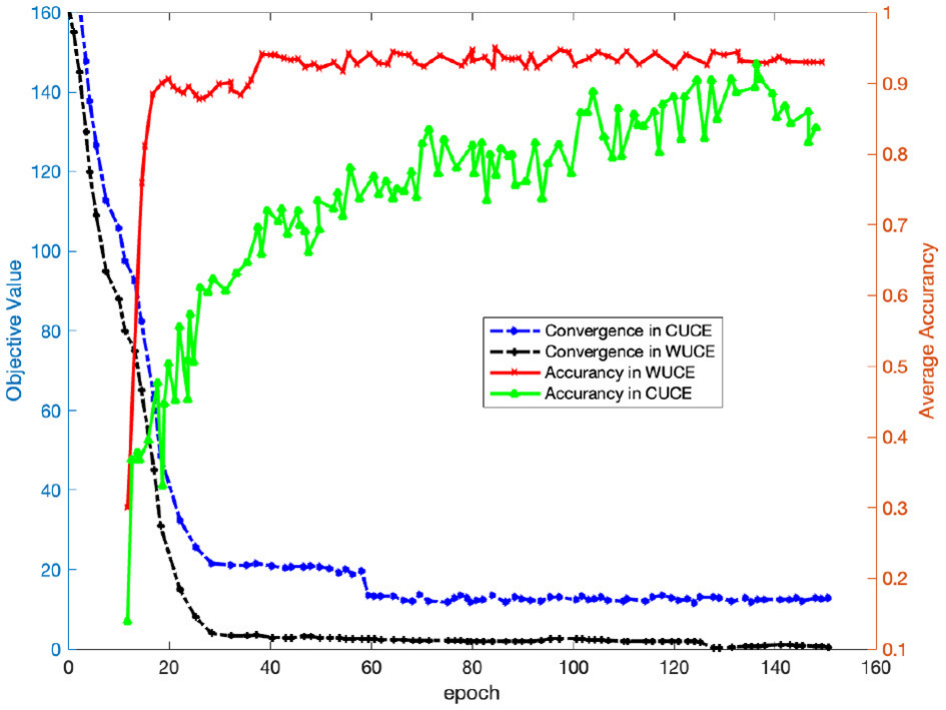
Convergence vs. accuracy on SEED.

It is crucial to evaluate the convergence of DADPc, since it is an iterative algorithm. Figure 5 presents the average experimental results obtained from three task protocols of the SEED dataset, with the right y-axis indicating the values. The curves in the figure illustrate that the proposed algorithm exhibits asymptotic convergence. Overall, the objective values of DADPc stabilize within 60 iterations. This trend was also evident in other recognition tasks with varying cross-session configurations.

Figure 5 depicts the recognition error on the left y-axis. WUCE outperforms CUCE in recognition accuracy due to the latter's complexity. Throughout the iterations, we observed notable improvements in recognition accuracy. Although there are some fluctuations in WUCE's accuracy when the number of iterations exceeds 40, the recognition accuracy generally remains above 90%. After 130 iterations, CUCE's recognition accuracy exceeds 80%.

#### 6.4.4 Effect of hyperparameters

**Figure 6:**
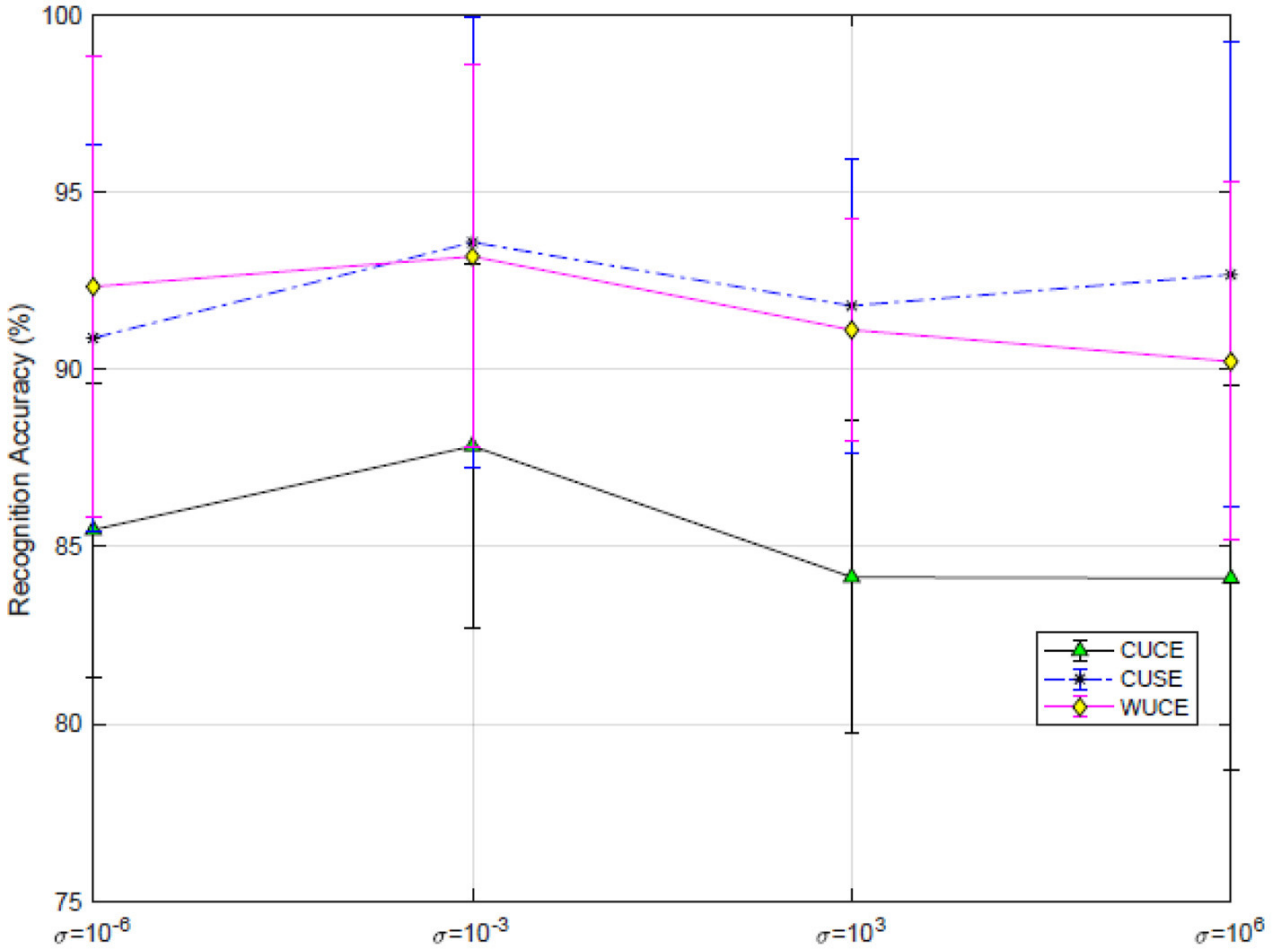
Effect of σ on SEED dataset.

As analyzed in Section 3.1 (Preliminary), the σ-norm approximates the *l*_2, 1_-norm as σ approaches 0 and converges to the Frobenius norm as σ tends to infinity. Figure 6 illustrates that the proposed method achieves optimal recognition performance at a σ value of 0.001 instead of these two extreme conditions. These results suggest that adaptively tuning σ during training could enhance the model's robustness by balancing sensitivity to outliers with feature representation fidelity.

**Figure 7:**
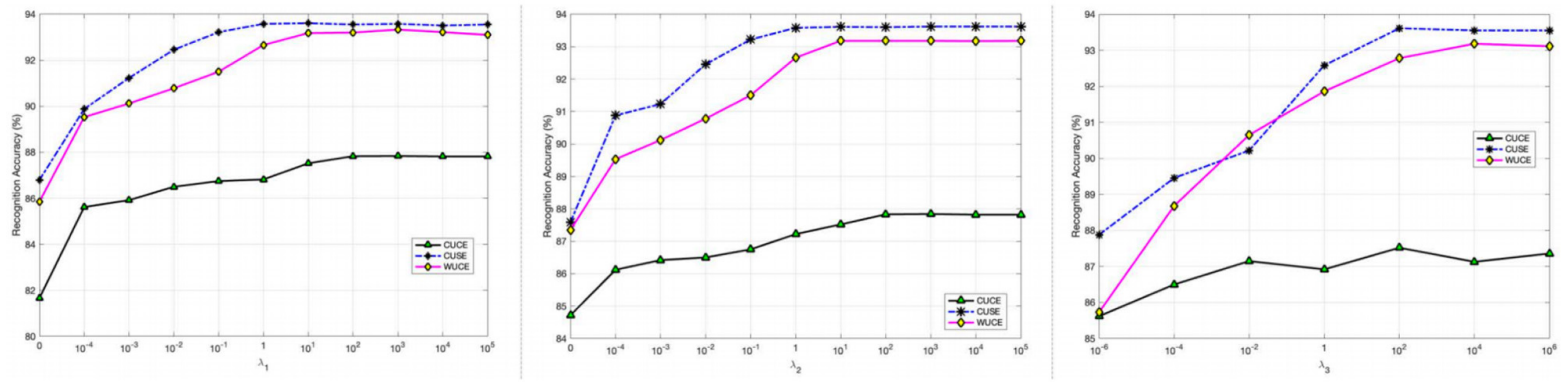
Effect of hyper-parameters on SEED dataset.

We analyze the impact of hyper-parameters λ_1_, λ_2_, and λ_3_ on the SEED datasets in Figure 7. The first subfigure in Figure 7 shows that performance varies with λ_1_. λ_1_ controls weight parameters. When λ_1_ is above 0, performance improves, peaking at λ_1_ = 100. The second subfigure in Figure 7 shows the best performance at λ_2_ = 1 or 10. As λ_1_ and λ_2_ near 0, performance drops, highlighting feature selection's role. The λ_1_ and λ_2_ prevent overfitting by regulating **W** and **b**, crucial for the small-sized SEED datasets. Finally, we study λ_3_, which regulates possibilistic clustering. The last subfigure in Figure 7 shows that when λ_3_ is 10 or higher, performance is stable. Tuning λ_3_ is difficult due to non-linearity (Nie et al., 2020). As λ_3_ approaches 0, the DADPc's performance exhibits a significant decrease, showing that possibilistic clustering mitigates noisy data and improves generalization. These results show that each hyperparameter contributes to DADPC's adaptability and requires careful tuning for optimal performance in the CUCE scenario.

#### 6.4.5 Ablation study

This ablation study systematically investigates the effectiveness of various components in the proposed model and presents the corresponding contributions of each component to the overall performance of the model DADPc. As shown in Table 8, when σ approaches 0, the adaptive norm of DADPc is the ℓ_2, 1_ norm, and as σ approaches ∞, the adaptive norm of DADPc becomes the Frobenius (F) norm. It can be observed that as σ decreases, the performances of both CUCE and WUCE improve to varying degrees, while the performance of CUSE slightly declines. A possible reason is that the data differences generated by different experimental objects across different sessions for CUCE and WUCE may be greater than the data generated by the same experimental objects in various sessions for CUSE. The ℓ_2, 1_ norm aids in feature selection, allowing it to identify more diverse and discriminative feature information from CUCE and WUCE, thus enhancing the model DADPc's discriminative effectiveness. However, a smaller σ does not always yield better results. A more detailed analysis of the hyper-parameter σ is provided in Section 6.4.5. Additionally, when λ_1_ = 0, the constraint on *W* is removed, which can lead to model overfitting and hinder further feature selection, resulting in a decline in the model's performance. When λ_2_ = 0, the constraint on *b* is removed, leading to only a slight decrease in the model's performance. Most importantly, when λ_3_ = 0, it is equivalent to removing the possibilistic clustering constraint term. In these three different experimental scenarios, the model's performance drops by about 20% to 30%. This phenomenon indicates that the possibilistic clustering constraint term significantly impacts improving DADPc's performance.

**Table 8:** The ablation accuracy(%) of our proposed model on SEED.

**Ablation strategy**	**CUCE**	**CUSE**	**WUCE**
DADPc with σ = 10^−8^(*l*_2, 1_-norm)	84.29 ± 4.37	90.24 ± 4.18	92.06 ± 6.25
DADPc with σ = 10^8^(*F*-norm)	83.20 ± 5.26	90.37 ± 3.62	90.10 ± 4.39
DADPc w/o constraints on **W**(λ_1_ = 0)	85.49 ± 6.39	91.60 ± 5.72	91.06 ± 7.11
DADPc w/o constraints on **b**(λ_2_ = 0)	87.21 ± 3.28	93.08 ± 6.72	92.88 ± 6.55
DADPc w/o PC(λ_3_ = 0)	63.43 ± 7.14	62.89 ± 6.82	75.69 ± 7.18
DADPc	**87.83** **±** **4.21**	**93.58** **±** **6.35**	**93.18** **±** **5.40**

#### 6.4.6 Spatial mapping of key EEG patterns for emotion recognition

**Figure 8:**
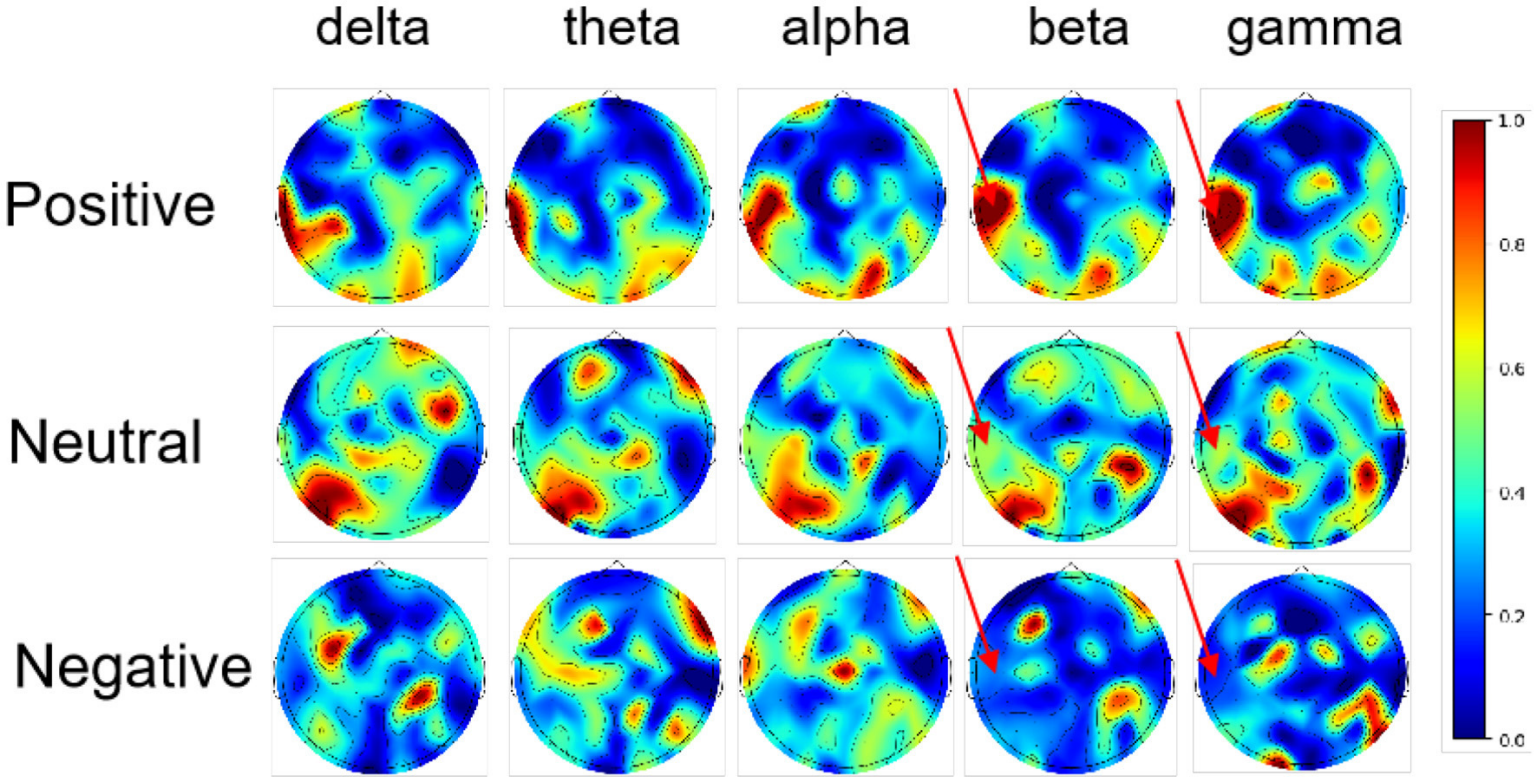
Topographic analysis of the mutual information between the EEG patterns and the model predictions.

During the spatial mapping process, we pinpoint crucial neural activities associated with emotion recognition by assessing the mutual information shared between these patterns and the predictive labels. More precisely, during the *i*th validation phase, we work with a dataset from the target domain, denoted as $X_{i}^{t}$, which is structured as an *N*_t_*310 matrix. Here, *N*_*t*_ represents the number of data points in the target domain, while 310 corresponds to the DE features extracted from 62 electrodes across five frequency ranges. The model's output predictions for this phase are represented by ${Ŷ}_{i}^{t}$ with a size of *N*_t_*3. Each column in ${Ŷ}_{i}^{t}$ represents the three emotions (positive, neutral, and negative) along with their corresponding prediction probabilities (Ross, 2014). The resultant mutual information matrix, designated as $I\left(X_{i}^{t},{Ŷ}_{i}^{t}\right)\in {\mathbb{R}}^{3\times 310}$, quantifies the intrinsic relationship between the EEG patterns and the model's predictions. This matrix $I\left(X_{i}^{t},{Ŷ}_{i}^{t}\right)$is then scaled to a range of [0,1], where higher values indicate a stronger contribution of the EEG patterns to the model's predictions during the *i*th validation phase. Figure 8 presents the average of all derived $I\left(X_{i}^{t},{Ŷ}_{i}^{t}\right)$ matrices across various validation phases. As illustrated in Figure 8, the mutual information in the region indicated by the red arrow for the beta and gamma frequency bands exhibits a color gradient from red to blue, where the color corresponds to emotional intensity. This region is responsible for visual processing and emotional regulation, and its high-frequency neural activity is associated with the perception of complex emotional stimuli. A darker red tone signifies more intense emotions, while a bluer tone indicates calmer or less intense emotional states, which aligns with the findings of Zheng and Lu (2015), particularly in the region indicated by the red arrow.

## 7 Conclusion

This study presents Domain Adaptive Deep Possibilistic Clustering (DADPc), a novel framework that unifies deep domain-invariant feature learning and possibilistic clustering to address key challenges in EEG-based emotion recognition: inter-subject variability, label scarcity, and noise sensitivity. By reformulating maximum mean discrepancy (MMD) as a one-centroid clustering task within a fuzzy entropy-regularized possibilistic framework, DADPc mitigates noise-induced domain shifts while enhancing feature discriminability. The integration of adaptive weighted loss and memory bank strategies further enhances pseudo-label reliability and cross-domain alignment. Extensive experiments on SEED, SEED-IV, and DEAP datasets demonstrate DADPc's superiority. However, the manual tuning of λ_3_ (fuzzy entropy weight) remains subjective, potentially limiting reproducibility across datasets (the last subfigure in Figure 7). Consequently, λ_3_ and *v*_*ik*_ constitute a direction deserving further exploration through Bayesian optimization or a meta-learning strategy.

## Data Availability

Publicly available datasets were analyzed in this study. This data can be found here: http://epileptologie-bonn.de/cms/upload/workgroup/lehnertz/eegdata.html and http://bcmi.sjtu.edu.cn/seed.
